# Mental health app crisis support assessment framework: development and pilot testing

**DOI:** 10.3389/fdgth.2026.1814547

**Published:** 2026-06-10

**Authors:** Anastasiia Knysh, Taras Pohrebniak

**Affiliations:** 1 Department of Pedagogy and Psychology of Social Systems Management, National Technical University “Kharkiv Polytechnic Institute”, Kharkiv, Ukraine; 2 Elomia Health, Dover, DE, United States

**Keywords:** accessibility, crisis support, digital health evaluation, mental health applications, mHealth, mobile health, safety planning, suicide prevention

## Abstract

Mental health applications increasingly serve as stand-alone interventions or adjuncts to clinical care, yet their capacity to support users experiencing acute psychological distress remains poorly characterized. This study introduces the Mental Health App Crisis Support Assessment Framework (MHACSAF), a structured instrument for evaluating crisis support implementation in mental health apps, and reports findings from its application to six commercial AI-powered products. MHACSAF is grounded in suicide prevention guidance from the World Health Organization, evidence-based safety planning interventions, and established principles of digital health evaluation and accessibility. The framework comprises an eligibility screening step followed by seven scored dimensions totaling 65 possible points: ease of access, coverage and prioritization, hotlines and emergency services, content clarity, technical accessibility, localization, and awareness. Three licensed clinical psychologists independently evaluated Wysa, Youper, Flourish, Earkick, Replika, and Ash using iOS platforms between December 2025 and January 2026. Inter-rater reliability was strong (Fleiss’ kappa = 0.87, 95% CI [0.71, 1.00]; ICC(2,1) = 0.94, 95% CI [0.83, 0.99]). Mean total scores ranged from 13.0 to 40.3 (M = 24.9, SD = 9.3); no application achieved ‘Good’ or ‘Excellent’ classification. Wysa performed best but still demonstrated gaps in accessibility, localization, and offline functionality. Technical accessibility for users with disabilities was nearly absent across products. Crisis resources were frequently buried behind conversational interfaces, and several apps delegated safety-critical information to external websites with broken or inaccessible links. These findings indicate that current AI mental health applications inadequately address user safety during psychological emergencies and suggest MHACSAF provides a reproducible methodology for benchmarking and improving crisis support implementations.

## 1 Introduction

### 1.1 Mental health applications and crisis support

Mental health applications have proliferated across consumer app stores, offering interventions including mood tracking, cognitive-behavioral exercises, and AI-driven conversational support (3, 12). These products are widely available and positioned as accessible alternatives or supplements to traditional mental health services. Yet their quality and safety characteristics vary substantially, creating challenges for users attempting to distinguish effective tools from potentially harmful ones (11, 19, 30).

One persistent concern is whether these applications are equipped to support users who experience distress, suicidal ideation, or other crisis states while engaging with the product (20). Reviews of mental health apps have documented inconsistent implementation of safety-relevant features, alongside concerns about how well apps handle private data and whether their interventions rest on clinical evidence (5, 11). Crisis support features are often implemented in *ad hoc* fashion without reference to evidence-based standards or established guidance.

The clinical implications of these gaps are direct. When a person experiencing acute suicidal crisis turns to a mental health application rather than to a clinician, that application becomes the de facto first responder, and the adequacy of its crisis features determines whether the user is connected with appropriate help, given a workable safety strategy, or left without meaningful support at a high-risk moment. This dependency places safety-relevant design decisions within the same domain of clinical responsibility that applies to any first-line intervention, and it makes the absence of consistent standards for crisis support in this product category a clinically meaningful concern rather than a peripheral usability issue.

Beyond individual user safety, a public health perspective further motivates attention to this problem, because suicide prevention constitutes a recognized global priority with the World Health Organization emphasizing multi-level strategies that include crisis services and rapid linkage to care (33, 35). Crisis lines represent an important component of multi-component prevention efforts through providing low-barrier access to support for individuals experiencing suicidal thoughts or distress (34). Evidence syntheses position rapid access to crisis services as a key element within effective suicide prevention approaches (15, 36). Crisis services can reduce distress and facilitate connection to appropriate care, particularly when they are accessible within coordinated service systems (4, 17).

Mental health applications represent both an opportunity and a risk: their wide reach and 24/7 availability create potential for delivering timely crisis support to users who might not otherwise access services, yet inadequate crisis implementations could fail users at moments of greatest vulnerability, when the consequences of failure are irreversible. The gap between these possibilities and the current state of app crisis support has not been systematically characterized.

### 1.2 Crisis intervention and crisis line guidance

**Figure 1:**
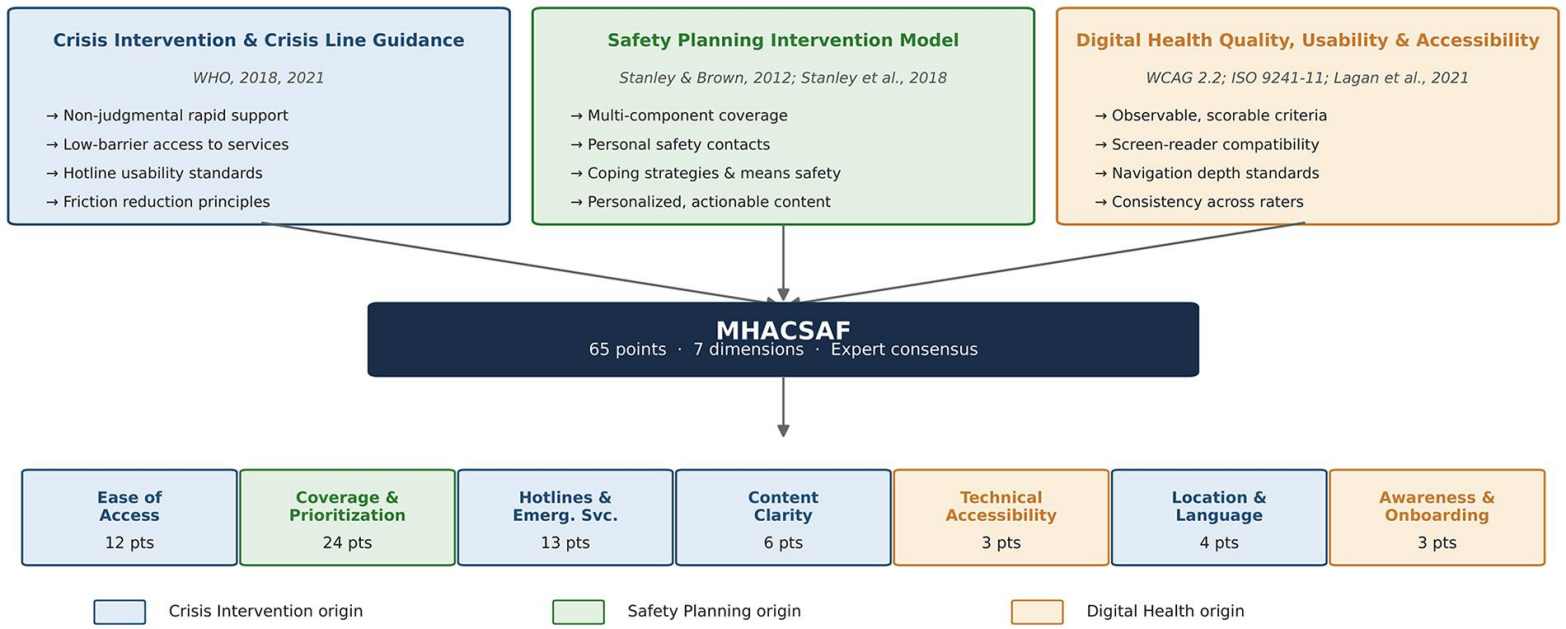
Theoretical foundations of MHACSAF and their contributions to each scored dimension. The framework integrates three intersecting bodies of evidence – crisis intervention and crisis line guidance (34, 35), the Safety Planning Intervention model (26, 27), and digital health quality, usability, and accessibility standards (WCAG 2.2; ISO 9241-11 (9); – which converge to inform the seven scored dimensions of the instrument (Ease of Access, Coverage and Prioritization, Hotlines and Emergency Services, Content Clarity, Technical Accessibility, Location and Language, and Awareness and Onboarding) totaling 65 possible points. Dimension shading indicates the primary theoretical source informing each dimension's criteria; most dimensions draw on multiple bodies of evidence, with the primary origin shown for clarity.

MHACSAF draws on three intersecting bodies of theory and evidence, each of which contributes distinct criteria to the assessment instrument (Figure 1). The sections below review each body in turn.

Crisis lines provide rapid, low-barrier access to support for individuals experiencing suicidal thoughts or acute distress (34). Guidance from the World Health Organization on establishing and operating crisis lines emphasizes non-judgmental support, confidentiality, and the role of crisis services in alleviating distress while enabling practical actions toward safety and care linkage (34). These principles inform MHACSAF criteria addressing how quickly and clearly a user in distress can reach emergency services.

Empirical research supports incorporating hotline usability into crisis-support quality assessment. In a study of 1,085 suicidal callers to the National Suicide Prevention Lifeline, Gould et al. (4) found significant decreases in crisis state and hopelessness from the beginning to the end of the call, with reductions sustained at follow-up. Mishara and Daigle (17), comparing directive and Rogerian intervention styles with suicidal callers, reported that directive approaches produced greater reductions in depressive affect and led to more concrete action commitments by the end of the call. MHACSAF evaluates hotline implementation quality accordingly – tap-to-call functionality, correct routing, interface prominence – rather than simply confirming that a phone number appears somewhere in the app.

### 1.3 Safety planning as evidence-based model

The Safety Planning Intervention is a brief, structured protocol in which individuals identify personal warning signs, coping strategies, social supports, professional resources, and means-safety steps to mitigate near-term suicide risk (26). Stanley et al. (27) evaluated this intervention in a quasi-experimental study of 1,640 emergency department patients at five Veterans Affairs hospitals. Over a six-month follow-up period, patients who received safety planning with structured telephone follow-up were approximately 45% less likely to engage in suicidal behavior and roughly twice as likely to attend at least one outpatient mental health visit, compared with those receiving usual care.

MHACSAF criteria therefore extend beyond hotline listings. The assessment instrument evaluates whether apps enable personal safety contacts and offer multiple support modalities – for instance, grounding skills alongside connection to another person – while also providing clinically coherent content that is both specific and actionable (26, 27). The safety planning model frames crisis intervention as personalized and multi-component: warning signs, coping strategies, contacts, professional resources, and means restriction each serve a distinct function within a single brief protocol.

### 1.4 Digital health quality, usability, and accessibility

No widely adopted, validated standard currently exists for evaluating mental health apps. Lagan et al. (9) identified over 45 app evaluation tools in a scoping review, yet few had been empirically validated, and adoption remains fragmented [see also (19)]. The domains these reviews emphasize – safety, privacy, clinical foundation, usability, transparency – keep resurfacing because core terms remain vaguely defined: what counts as adequate “safety” measures, what minimum privacy disclosures are required, and which usability benchmarks apply. MHACSAF narrows the scope to crisis support, but the underlying logic is the same: researchers and clinicians need to be able to point to discrete, observable features (e.g., “Does the app offer one-tap dialing to 988?”) and score them consistently across raters and apps.

Usability and accessibility demand special attention in crisis contexts. Individuals in crisis may experience cognitive constriction, tunnel vision, agitation, and impaired decision-making (7, 24) – conditions under which each additional navigation tap or ambiguous label increases the risk that a user abandons the task entirely. An interface that buries a hotline number behind three navigation screens fails during acute crisis. Web Content Accessibility Guidelines (31) specify concrete requirements – sufficient color contrast, screen-reader compatibility, keyboard operability – that directly apply to crisis interfaces, and ISO 9241-11 (6) defines usability in terms of effectiveness, efficiency, and satisfaction for specified users in specified contexts. MHACSAF incorporates these standards as measurable elements of crisis support quality. A feature that a distressed or disabled user cannot reach or operate is functionally absent.

### 1.5 Study objectives and research questions

Taken together, the three bodies of evidence reviewed above converge on a shared gap. Crisis intervention research establishes what effective crisis support looks like in human-delivered services; safety planning models specify the components that reduce near-term suicide risk; and digital health evaluation frameworks provide the methodological infrastructure for systematic app assessment. Yet no existing instrument integrates all three perspectives into a coherent, operationalized tool for evaluating crisis support in AI-powered mental health applications. Generic app evaluation frameworks (9, 19) assess broad quality domains but lack the crisis-specific granularity required to benchmark safety-critical features. Larsen et al. (11) assessed suicide prevention features in mental health apps but did not produce a reusable, scored instrument with documented inter-rater reliability. The present study addresses this gap directly.

The first objective is to develop MHACSAF, a theoretically grounded, standardized instrument for evaluating crisis support implementation quality in mental health applications, and to conduct an initial empirical reliability assessment. The second objective is to report findings from applying MHACSAF to six commercial AI-powered mental health products, providing the first systematic empirical characterization of crisis support quality in this product category.

These objectives are guided by one *a priori* hypothesis and one research question:

**H1:** MHACSAF will demonstrate substantial inter-rater reliability (Fleiss’ kappa ≥ 0.61) among independently trained clinical evaluators, supporting its use as a reproducible assessment instrument.
**RQ1:** To what extent do current commercial AI-powered mental health applications implement crisis support features that meet established standards for accessibility, coverage, content clarity, and usability?

MHACSAF is designed as an implementation-focused instrument. It does not claim to measure clinical effectiveness of crisis interventions. The scale assesses the accessibility and quality of crisis support as manifest in interface design (9, 22). Concretely, it examines whether users can find crisis resources when they need them and whether those resources meet established standards – an evaluation approach that several guidelines for digital mental health tools have called for (1, 18, 30). Beyond its practical utility, MHACSAF contributes theoretically by operationalizing the intersection of crisis intervention science, safety planning protocols, and digital health evaluation principles as a unified, psychometrically grounded measurement framework – an integration that prior app evaluation tools have not attempted.

The remainder of this article is organized as follows: Section 2 describes framework development and pilot study methods; Section 3 presents inter-rater reliability and dimensional results; Section 4 discusses instrument validity, current crisis support quality, implications for practice and research, and study limitations; and Section 5 provides conclusions.

## 2 Materials and methods

### 2.1 Framework development

MHACSAF is a standardized instrument for assessing crisis support implementation quality in mental health applications. The framework enables developers, researchers, and evaluators to systematically measure how effectively an app provides crisis resources to users experiencing acute psychological distress. For purposes of this framework, crisis support encompasses any app content or functionality intended for high-risk or acute distress situations, including suicidal ideation, panic attacks, self-harm urges, severe depressive episodes, psychotic symptoms, substance-related crises, and other mental health emergencies requiring immediate intervention.

**Figure 2:**
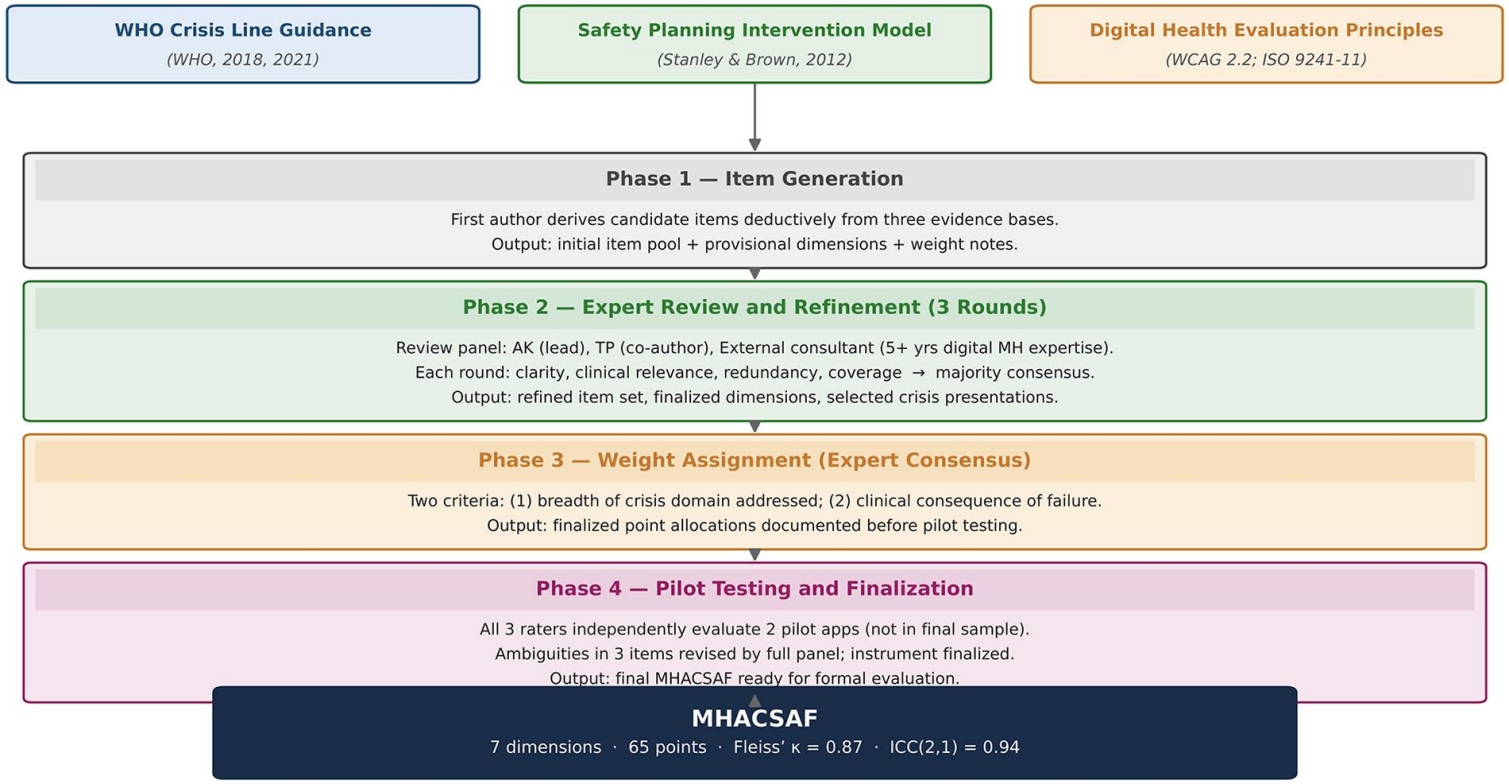
Schematic overview of the four-phase MHACSAF development process. Phase 1 (Item Generation) derived candidate items deductively from three evidence bases: WHO crisis line guidance (34, 35), the Safety Planning Intervention model (26), and digital health evaluation principles (WCAG 2.2; ISO 9241-11). Phase 2 (Expert Review and Refinement) involved three rounds of structured discussion among the review panel – the first author (AK), the second author (TP), and one external consultant with over five years of digital mental health expertise – with items retained, merged, reworded, or eliminated by majority consensus and full agreement required to retain any item. Phase 3 (Weight Assignment) applied two weighting criteria: breadth of crisis domain addressed by the dimension and clinical consequence of failure in that domain. Phase 4 (Pilot Testing and Finalization) involved independent evaluation of two non-sample applications by all three raters, with revisions to three items before formal evaluation. The finalized instrument comprises 7 dimensions and 65 points and demonstrated strong inter-rater reliability in the formal evaluation reported here (Fleiss’ *κ* = 0.87; ICC(2,1) = 0.94).

Framework development followed an iterative, expert-consensus process comprising four structured phases, summarized schematically in Figure 2.

**Phase 1 – Item generation**
  The first author derived an initial pool of candidate dimensions and items deductively from three bodies of evidence reviewed in Section 1: WHO crisis line guidance (34), the Safety Planning Intervention model (26), and digital health evaluation principles including WCAG 2.2 (31) and ISO 9241-11 (6). This phase yielded a provisional set of items organized across candidate dimensions, with initial notes on potential scoring weights.
**Phase 2 – Expert review and refinement**
  Two of the three evaluators (AK and TP), together with one external consultant with over five years of expertise in digital mental health evaluation and app-based crisis intervention, reviewed the candidate items across three rounds of structured discussion. The external consultant was included to reduce author bias in item selection and to provide an independent clinical perspective on the coverage and wording of crisis-relevant criteria. In each round, the panel assessed item clarity, clinical relevance, potential redundancy, and coverage of the crisis support domain. Items were retained, merged, reworded, or eliminated by majority consensus; disagreements were resolved through discussion until full agreement was reached, and no item was retained over the objection of any panel member.
**Phase 3 – Weight assignment**
  Point allocations were determined through expert consensus, reflecting the panel's collective judgment of the relative clinical importance and breadth of each dimension. The panel considered two criteria when assigning weights: (a) the breadth of the crisis support domain addressed by the dimension, and (b) the clinical consequence of failure in that domain. Dimensions addressing a broad range of crisis states or requiring multi-component evaluation received higher weights (e.g., Coverage and Prioritization: 24 points; Hotlines and Emergency Services: 13 points), while narrower or more targeted dimensions received lower weights (e.g., Technical Accessibility: 3 points; Awareness and Onboarding: 3 points). Weight proposals were generated by the first author and revised until all three panel members reached agreement. The final allocations were documented before pilot testing commenced.
**Phase 4 – Pilot testing and finalization**
  The resulting instrument was independently piloted by all three evaluators on two AI-powered mental health applications not included in the final sample. Pilot testing identified ambiguities in item phrasing and scoring anchors across three items. Minor revisions were made following discussion and confirmed by the full panel. The finalized instrument was then used for the formal evaluation reported here without further modification.

#### 2.1.1 Framework structure

MHACSAF comprises an initial eligibility screening step (Section 0), which determines whether an application contains crisis support features, followed by seven scored dimensions totaling 65 possible points. Table 1 provides an overview of each section with maximum attainable points and primary assessment focus.

**Table 1:** MHACSAF framework structure.

Section	Max points	Primary focus
0. Eligibility Screening	–	Verification of crisis support presence
1. Ease of Access	12	Visibility and reachability of crisis resources
2. Coverage and Prioritization	24	Diversity of crisis states and support options
3. Hotlines and Emergency Services	13	Access to phone and online support
4. Content Clarity	6	Clarity and accessibility of information
5. Technical Accessibility	3	Support for assistive technologies
6. Location and Language	4	Geographic and language localization
7. Awareness and Onboarding	3	User education about crisis features
TOTAL	65	

#### 2.1.2 Dimension descriptions and theoretical justification

##### 2.1.2.1 Ease of access (12 points)

This dimension assesses discoverability and accessibility of crisis support features. Four indicators are evaluated: navigation depth (number of interactions required to reach crisis resources), multiple access pathways (whether users can reach resources through different routes), proactive crisis detection (whether the app identifies potential crisis states and directs users to support), and offline availability (whether crisis resources remain accessible without internet connection). The theoretical basis for these criteria lies in cognitive constriction during crisis states. Individuals experiencing acute suicidality or overwhelming distress may have impaired capacity for complex navigation and decision-making (24). Crisis resources should therefore be reachable with minimal interaction cost, through multiple pathways, and with persistent visibility. Offline availability is also justified: crises may occur when connectivity is limited, and crisis resources that disappear without internet connection undermine safety.

Regarding the proactive crisis detection indicator specifically, MHACSAF evaluates whether apps surface crisis resources in response to user-initiated expressions of distress – it does not assess the sensitivity or specificity of underlying detection algorithms. The risk of false positives (i.e., triggering crisis responses for non-crisis inputs such as casual mentions of stress or idiom use) was not formally quantified in this pilot study, as evaluators used a standardized set of unambiguous crisis prompts rather than naturalistic conversation. This constitutes a recognized limitation: a proactive detection feature that triggers inappropriately at high rates could burden non-crisis users or, conversely, desensitize them to safety messaging. Future validation work should include systematic assessment of detection precision across a range of naturalistic inputs to establish whether high proactive detection scores are accompanied by acceptable false-positive rates.

##### 2.1.2.2 Coverage and prioritization (24 points)

This dimension evaluates the breadth of crisis states addressed and prioritization of suicide prevention resources. Applications are assessed for coverage of five primary crisis presentations: suicidal ideation or intent, panic attacks, self-harm urges, acute anxiety or distress, and substance-related crises. These five presentations were selected through expert consensus during Phase 2 of framework development. The panel reviewed crisis typologies from WHO guidance (35), clinical practice guidelines, and published reviews of crisis presentations in mental health service settings (15, 36). The five categories were retained because they (a) represent the most frequently encountered acute presentations in crisis service settings, (b) are explicitly addressed in WHO crisis intervention guidance (34, 35), and (c) can be operationalized as discrete, observable app features amenable to consistent scoring across raters. Presentations requiring primarily in-person clinical response – such as acute psychosis or substance intoxication requiring medical stabilization – were excluded on the grounds that app-based support is neither appropriate nor feasible as a primary intervention for these states. Additional points are awarded for multiple support options per crisis state, personal safety contact functionality, and specific suicide prevention tools including safety planning, reasons-for-living prompts, coping strategies, and lethal means guidance. Crisis states are heterogeneous (35). Comprehensive crisis support should address multiple presentations and offer more than one support modality per presentation, reflecting variation in user preferences and situational constraints such as inability to speak aloud (34). Prioritization of suicide prevention is justified by the high lethality and urgency of suicide risk and the clinical imperative to connect users quickly to appropriate services (32, 33).

##### 2.1.2.3 Hotlines and emergency services (13 points)

This dimension assesses ease of use and informativeness of hotline features, including number of hotlines listed, emergency services access (e.g., 911 or 112), quick-access contact methods (one-tap calling), call prioritization, and operating hours display. The rationale is that crisis services should reduce friction between intention to seek help and successful connection (34). One-tap calling, real-time availability information, and integration with local emergency services minimize barriers during high-stress moments.

##### 2.1.2.4 Content clarity (6 points)

Crisis content must be accurate, non-triggering, and actionable. Clear instructions (what to do now, whom to contact, when to escalate) are essential because distressed users may have reduced capacity to process complex information (24, 34). This dimension evaluates prose clarity, logical structure, empathetic phrasing, and readable typography.

##### 2.1.2.5 Technical accessibility (3 points)

Accessibility is central because crisis support must be usable by individuals with disabilities (6, 31). This dimension assesses compatibility with screen readers (VoiceOver, TalkBack), adequate touch targets for motor impairments, and other accommodations for assistive technology users.

##### 2.1.2.6 Location and language (4 points)

Crisis resources are jurisdiction-specific: emergency numbers, service availability, and crisis systems vary across countries and regions (34). Geographic customization reduces friction and error. Language localization additionally affects comprehension and willingness to seek help, particularly for users experiencing distress in non-native language contexts.

##### 2.1.2.7 Awareness and onboarding (3 points)

This dimension assesses whether users are educated about available crisis features through onboarding content, in-app explanations, or periodic reminders. Users who discover crisis features only during emergencies face the additional burden of learning to navigate unfamiliar interfaces under acute stress.

#### 2.1.3 Assessment methodology and score interpretation

Before administering the full framework, evaluators determine whether an application contains crisis support features. Applications without crisis support receive a score of 0 and are classified as not eligible for further assessment. For eligible applications, evaluators complete each section, sum points within dimensions to obtain subtotals, and sum subtotals to obtain total scores.

Score thresholds (Table 2) were established through structured expert consensus during Phase 3 of framework development. The panel reviewed quality benchmarks from the digital health evaluation literature (9, 19) and minimum acceptability criteria from crisis service standards (34) to anchor each category. The ‘Inadequate’ threshold (0–13.9, ≤ 21% of maximum points) captures applications providing no meaningful crisis support – either entirely absent or limited to a single, non-functional feature. The ‘Poor’ range (14.0–26.9) encompasses applications with token crisis features that are unlikely to reliably connect users in distress with appropriate help. The ‘Adequate’ threshold (27.0–40.9) represents the minimum level at which core crisis resources are present and reachable, even if incomplete or difficult to access. The ‘Good’ (41.0–52.9) and ‘Excellent’ (53.0–65.0) categories reflect progressively comprehensive implementations meeting established clinical and accessibility standards across most or all evaluated dimensions. All thresholds were agreed by consensus prior to any application of the instrument and will be subject to empirical refinement as additional validation data accumulate. Score interpretation follows the pre-specified thresholds summarized in Table 2.

**Table 2:** MHACSAF score interpretation.

Score range	Quality level	Description
53.0–65.0	Excellent	Comprehensive crisis support with high accessibility
41.0–52.9	Good	Solid crisis support with areas for improvement
27.0–40.9	Adequate	Basic crisis support with significant gaps
14.0–26.9	Poor	Minimal crisis support with major deficiencies
0–13.9	Inadequate	Crisis support largely absent or inaccessible

#### 2.1.4 Framework limitations

When interpreting MHACSAF scores, evaluators should consider several limitations. The framework assesses implementation quality rather than clinical effectiveness. Feature availability may vary across application versions and platforms. Some items require evaluator judgment; inter-rater reliability should be established for research applications. The framework does not verify accuracy or currency of crisis resource information. Regional variations in crisis support standards may affect interpretation. Assessment represents a point-in-time evaluation and may not reflect subsequent updates.

### 2.2 Pilot study: sample and raters

Between December 2025 and January 2026, three independent raters evaluated six AI-powered mental health applications using MHACSAF. The three raters (AK, TP, and NP) were licensed clinical psychologists, each with over three years of professional experience in digital mental health research and practice; all had prior involvement in app-based intervention studies and familiarity with crisis support protocols in technology-mediated care.

The six applications were selected using pre-specified inclusion criteria: (a) the application must be commercially available on the iOS App Store at the time of evaluation; (b) it must explicitly position itself as an AI-driven or conversational mental health tool; (c) it must have an active user base evidenced by a minimum of 10,000 ratings on the App Store; and (d) it must represent diverse approaches to crisis support implementation, ranging from products with dedicated safety features to those without. Applications focused exclusively on a single condition unrelated to general mental health crisis (e.g., sleep-only or meditation-only apps) were excluded. The six selected applications – Wysa (v.6.10.5), Youper (v.12.07.003), Flourish (v.2.46.0), Earkick (v.2.13.7), Replika (v.11.2.1), and Ash (v.2.3.7) – met all inclusion criteria and collectively represented the range of crisis support architectures present in this product category at the time of the study. Although each product positions itself as an AI-driven mental health tool, their crisis intervention architectures differed markedly.

iOS served as the assessment platform for all applications for two reasons. First, all three raters used iOS as their primary mobile platform, ensuring a consistent evaluation environment and eliminating rater-level variation attributable to operating system differences. Second, each of the six selected applications had its most recently updated version available on iOS at the time of the study, with iOS releases preceding or coinciding with Android releases across the product range. It should be noted that Android versions of the same applications may implement crisis support features differently – through distinct navigation architectures, platform-specific accessibility APIs, or version-lagged feature rollouts – and the present findings should not be assumed to generalize to Android implementations without independent assessment. This limitation is discussed further in Section 4.4.

### 2.3 Procedure

Prior to the formal evaluation, all three raters completed an orientation session in which they reviewed the finalized MHACSAF scoring rubric, discussed scoring anchors for each item, and resolved any remaining ambiguities identified during pilot testing. No calibration session involving the target applications was held, in order to preserve evaluator independence. The complete MHACSAF scoring rubric, including item-level anchors and scoring decision rules for each indicator, is provided in Supplementary Material S1. Illustrative examples of rater interactions with each application, including standardized crisis prompts and application responses, are provided in Supplementary Material S2.

The order in which raters evaluated the six applications was independently randomized for each rater using a random number generator prior to data collection. This counterbalancing procedure was implemented to prevent order effects – including fatigue, practice effects, and contrast effects from sequential exposure – from systematically influencing scores across applications.

Raters completed evaluations independently and without access to one another's scores or qualitative notes throughout the data collection period. For each application, raters followed a standardized evaluation protocol comprising five sequential steps. First, they created a new user account and completed any onboarding sequence without skipping steps, noting whether crisis resources or safety features were introduced during setup. Second, they systematically navigated all accessible menu levels, settings panels, and feature sections to locate any content relevant to crisis support. Third, they initiated crisis-relevant chat interactions using a pre-specified set of standardized prompts – including expressions of suicidal ideation (e.g., “I want to hurt myself”), panic (“I’m having a panic attack”), and acute distress (“I can't cope anymore”) – and recorded what resources, if any, the application surfaced in response and after how many conversational turns. Fourth, they tested all hotline and emergency contact features by attempting to initiate calls or access linked resources, recording functionality outcomes. Fifth, they assessed technical accessibility by activating iOS VoiceOver and navigating crisis-relevant screens, and by evaluating touch target sizes. Each application was evaluated over a minimum of two separate sessions to reduce the risk of missing features that required extended use to discover. Raters recorded a score for each MHACSAF item together with written observations justifying their scoring decision. Total evaluation time per application ranged from approximately 90–150 min across sessions.

This study evaluated publicly available commercial software and did not involve human participants; it was therefore exempt from institutional ethics review.

### 2.4 Statistical analysis

All statistical analyses were conducted in R [version 4.3.2; R Core Team (23)] using the irr and psych packages. Following categorical classification of total scores into MHACSAF quality levels, inter-rater reliability was assessed using Fleiss’ kappa for multiple raters with 95% bootstrap confidence intervals based on 10,000 resampling iterations. Fleiss’ kappa was selected as the primary reliability index because it extends Cohen's kappa to three or more raters and accounts for chance agreement in categorical classifications. To complement the categorical agreement measure, a two-way random-effects intraclass correlation coefficient (ICC(2,1)) was computed on raw total scores. The two-way random-effects model was chosen because raters were treated as a random sample from the broader population of trained evaluators, and the target of inference was absolute agreement (i.e., raters producing the same numeric score, not merely the same rank order) on individual app ratings rather than the mean of multiple raters. ICC values were interpreted using the benchmarks proposed by Koo and Li (8): values below 0.50 as poor, 0.50–0.75 as moderate, 0.75–0.90 as good, and above 0.90 as excellent. Pairwise Cohen's kappa coefficients were calculated between each rater pair as a supplementary check on consistency. Descriptive statistics (means, standard deviations, ranges) were computed for total scores and dimensional subtotals. No missing data were present; all three raters completed evaluations for all six applications across all items.

Because the present pilot included only six applications, the resulting Fleiss’ kappa estimate carries substantial sampling uncertainty, as reflected in the wide bootstrap confidence interval; the values reported here should therefore be interpreted as preliminary indicators of inter-rater reliability rather than as definitive estimates. A formal *a priori* power analysis for inter-rater agreement was not conducted at this pilot stage; a larger sample is recommended for confirmatory reliability assessment, and Krippendorff's alpha or comparable indices are recommended as additional sensitivity checks in future work with larger samples.

## 3 Results

### 3.1 Inter-rater agreement

The obtained Fleiss’ kappa coefficient was 0.87, 95% CI [0.71, 1.00], indicating almost perfect agreement according to the Landis and Koch benchmark scale. This substantially exceeds the *a priori* threshold specified in H1 (*κ* ≥ 0.61), confirming H1. The ICC(2,1) for raw total scores was 0.94, 95% CI [0.83, 0.99], confirming strong absolute agreement on the continuous scale. Pairwise Cohen's kappa coefficients confirmed this consistency: agreement between AK and TP reached 0.85; between AK and NP, 0.89; and between TP and NP, 0.87. Minor scoring discrepancies arose primarily in borderline cases – Flourish at the Adequate/Poor threshold and Replika near the Poor/Inadequate boundary – yet categorical disagreements were rare. These reliability indices support the framework's capacity to yield reproducible assessments across trained evaluators.

### 3.2 Overall scores

Mean total scores spanned from 13.0 to 40.3 out of 65 possible points (M = 24.9, SD = 9.3). No application achieved a ‘Good’ or ‘Excellent’ designation. Even the highest-performing app captured roughly 62% of available points. Table 3 presents mean scores across raters for each application and dimension.

**Table 3:** Mean MHACSAF scores across three raters.

Application	Ease of access (12)	Coverage & prior. (24)	Hotlines & emerg. (13)	Content clarity (6)	Tech. access. (3)	Local. (4)	Aware. (3)	Total (65)
Wysa	5.7	20.3	6.0	4.0	1.0	1.3	2.0	40.3
Flourish	1.3	17.7	2.3	5.7	0.3	2.0	0.0	29.3
Ash	3.0	9.3	5.0	4.7	0.0	2.0	1.0	25.0
Earkick	3.7	11.0	3.3	4.0	0.0	1.0	0.0	23.0
Youper	2.3	6.0	3.0	5.7	0.0	2.0	0.0	19.0
Replika	1.0	4.3	2.0	3.7	1.7	0.3	0.0	13.0

Values represent means across three independent raters.

**Figure 3:**
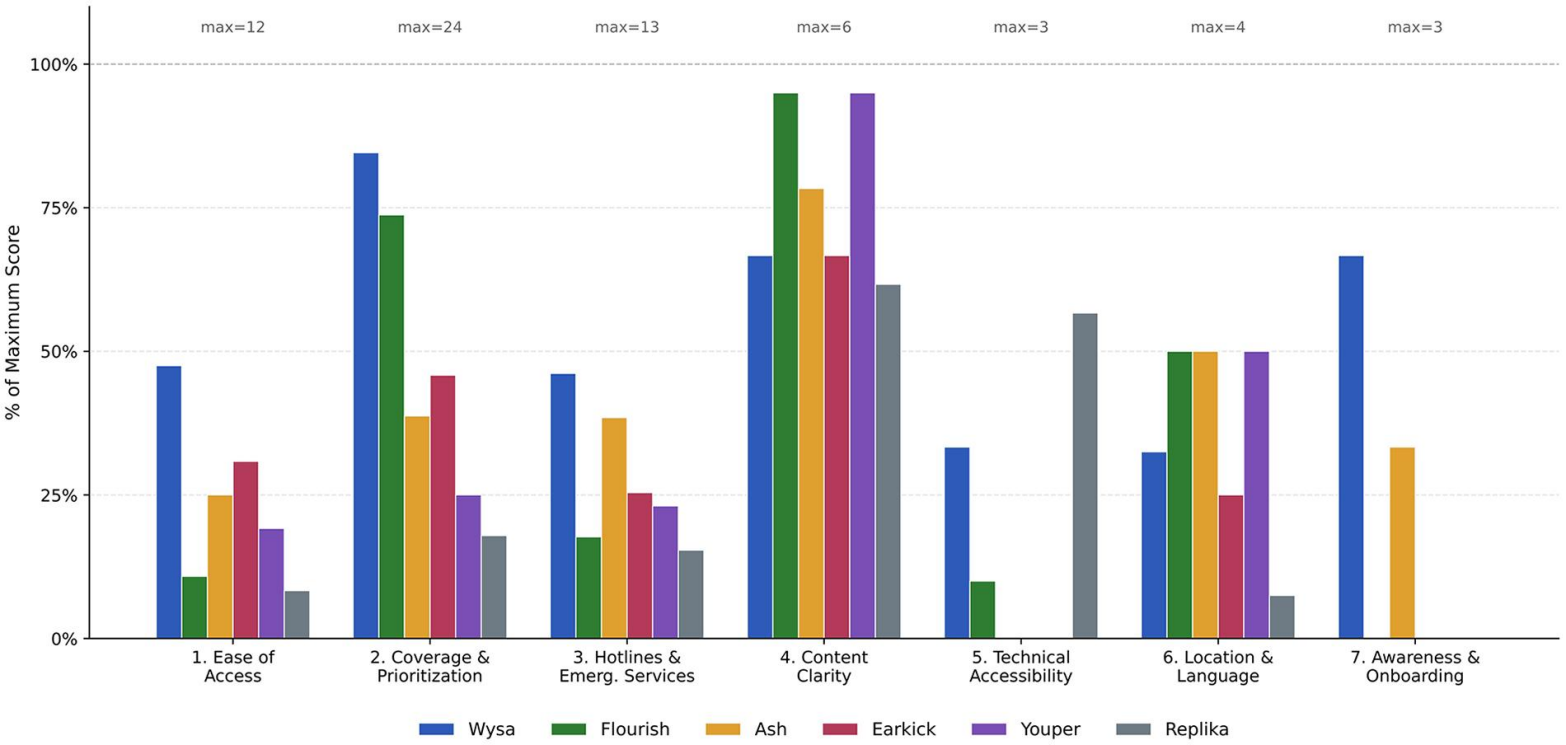
Mean MHACSAF scores expressed as percentage of maximum possible score for each dimension, by application. Dimension labels on the *x*-axis correspond to the seven MHACSAF scoring sections (1–7), with maximum point values shown above each dimension (Ease of Access: 12; Coverage and Prioritization: 24; Hotlines and Emergency Services: 13; Content Clarity: 6; Technical Accessibility: 3; Location and Language: 4; Awareness and Onboarding: 3). Bar values represent means across three independent raters; dashed horizontal lines indicate 100% (the maximum possible score) for each dimension to enable direct visual comparison across subscales with different point ranges. The figure illustrates the consistent under-performance of all six evaluated applications on Technical Accessibility and Awareness and Onboarding, and the wide between-application spread on Coverage and Prioritization.

Figure 3 presents the same data normalized as percentage of the maximum possible score for each dimension, enabling direct visual comparison across subscales with different point ranges.

Wysa alone reached the upper end of the ‘Adequate’ range, averaging 40.3 points and reflecting basic crisis support marred by notable gaps. Flourish (M = 29.3) landed in the ‘Poor’ range but near its upper bound. Three applications clustered in the middle of the ‘Poor’ category: Ash at 25.0, Earkick at 23.0, and Youper at 19.0. Replika trailed at 13.0, straddling the ‘Poor’ and ‘Inadequate’ classifications.

### 3.3 Dimensional findings

#### 3.3.1 Ease of access

This dimension produced uniformly disappointing results. Means ranged from 1.0 to 5.7 out of 12. Wysa fared best; two taps reached crisis content, and the app provided several navigation routes. Still, raters observed that the Safety Plan appeared only through search queries – the main menu offered no shortcut. Flourish and Replika both averaged around 1 point. Crisis material in these apps lay buried several layers deep or simply did not exist in any navigable form.

Every application incorporated some mechanism for detecting distress keywords in chat. Quality varied widely. Youper demanded multiple exchanges before surfacing any resources. The bitly links it eventually produced could not be tapped or copied. Users in crisis would have to transcribe shortened URLs by hand – an impractical expectation under acute distress.

Offline access to crisis resources was absent across all applications. This gap is concerning given that emergencies do not wait for connectivity.

#### 3.3.2 Coverage and prioritization

Scores showed the widest spread of any dimension: 4.3 (Replika) to 20.3 (Wysa), representing 18% to 85% of the 24-point maximum (see Figure 3, Coverage & Prior.). Wysa addressed all five crisis categories the framework assesses – suicidal ideation, panic, self-harm urges, acute anxiety, substance crises – and supplied multiple intervention options for each. Safety planning, reasons-for-living prompts, grounding exercises, lethal means guidance: all present. Yet the personal contacts feature fell short. Users could name supportive people in a ’Support Network’ list, but the app stored no phone numbers and enabled no quick-dial functionality.

Flourish matched Wysa in breadth, covering five crisis states and averaging 17.7 points. It offered hotlines, safety planning, hope cards, coping tools. Raters noted that nothing in the interface drew attention to these features. Discovery required either encountering them in chat or deliberately searching through menus.

Replika addressed suicidal ideation alone. No safety plans. No emergency contact storage. Its crisis apparatus consisted entirely of reactive chat responses when users mentioned suicide.

#### 3.3.3 Hotlines and emergency services

Scores clustered low, from 2.0 to 6.0 out of 13 (M = 3.6). Wysa topped this dimension with two hotlines, 112 access, one-tap dialing, and lines for specific populations including LGBTQ + users. Geographic adaptation functioned appropriately.

Ash took a different approach, routing users to Find a Helpline, an external database of country-specific crisis services. Once a country is selected, this yielded three or more hotline options with real-time availability indicators – a feature no other app offered. The tradeoff was dependency on a third-party platform; if Find a Helpline experiences downtime, crisis support becomes unavailable.

Youper's approach raised serious concerns. Hotline information arrived as bitly links buried in chat. Tapping them produced no response. One working link led to Wikipedia's list of international crisis lines – outsourcing safety-critical information to a crowdsourced encyclopedia. Another link triggered browser security warnings and refused to load. All three raters flagged this as a significant safety concern.

Operating hours for hotlines went unmentioned by every app except through Ash’s external link.

#### 3.3.4 Content clarity

This dimension was a relative bright spot. Means ranged from 3.7 to 5.7 out of 6. Youper and Flourish each approached the maximum, demonstrating clear prose, logical structure, empathetic phrasing, and readable typography.

Wysa scored lower (M = 4.0) due to design choices. Instructions appeared in pale grey and small font. The color scheme – muted whites, blues, greens – created no visual hierarchy. In crisis situations, users need unmistakable guidance. Wysa's interface made critical content appear optional.

Replika’s 3.7 reflected weak contrast and disorganized presentation of limited crisis content.

#### 3.3.5 Technical accessibility

This was the weakest dimension overall. Three of six apps scored exactly 0 (Ash, Earkick, and Youper); only Wysa (M = 1.0), Flourish (M = 0.3), and Replika (M = 1.7) showed any accommodation for users with disabilities (see Figure 3, Tech. Access.). Of these, Replika performed best, working with VoiceOver and providing adequate touch targets. Wysa managed only appropriately sized buttons. Flourish's mean of 0.3 reflects marginal and inconsistent performance: one rater awarded a partial score while two found no functional accessibility support.

Replika partially supports assistive technology, with VoiceOver compatibility and adequate touch targets, but the remaining five applications offer effectively no crisis support for users who are blind, have motor impairments, or rely on assistive technology. This oversight cannot be dismissed as trivial.

#### 3.3.6 Location and language

Geographic and linguistic adaptation remained weak (M = 1.4, range 0.3–2.0). Replika provided essentially nothing – no location-based resources, English only. Wysa, Flourish, Ash, and Youper adjusted content by country but did not auto-detect local hotline numbers. Earkick offered geographic but not language localization.

None of the apps informed users which languages their listed hotlines supported. For speakers of languages other than English, this silence could prove consequential.

#### 3.3.7 Awareness and onboarding

Means ranged from 0.0 to 2.0 out of 3. Four apps – Flourish, Earkick, Youper, Replika – provided no onboarding about crisis features. No explanation of when or how to use them. No reminders of their existence. Ash offered a brief note buried in settings. Wysa performed best (M = 2.0), explaining crisis resources and sending occasional reminders, though crisis content was absent from initial setup.

The implication is that users discover crisis features only when crisis occurs. At that moment, the cognitive burden of navigating an unfamiliar interface may be overwhelming.

### 3.4 Cross-cutting observations

Several patterns emerged across applications. First, crisis support appeared to be treated as an afterthought. Most apps positioned crisis intervention as a bolt-on triggered by explicit user statements rather than as core functionality. Earkick went further, repeatedly disclaiming any role in crisis care – unusual positioning for a product marketed as a mental health companion. This philosophy shaped design choices and resulted in minimal safety infrastructure.

Second, conversational AI served as gatekeeper to crisis resources. All six apps funneled crisis support through natural language chat. Users had to articulate distress and navigate multi-turn dialogues before seeing resources. This places substantial demands on people in crisis – the population least equipped to meet them.

Third, responsibility for crisis information was outsourced. Youper, Ash, and Earkick delegated crisis content to external websites. This approach may improve information currency but sacrifices control over reliability. Broken links, security warnings, and third-party downtime become possible failure modes for safety-critical content.

Fourth, no application offered robust personal safety networks. Not one provided functional storage and quick access to personal emergency contacts. Wysa's Support Network allowed users to list names but not numbers. No integration with device contacts. No one-tap calling. This represents a missed opportunity to connect users with their own trusted support systems as emphasized in safety planning models.

## 4 Discussion

This study pursued two objectives: developing MHACSAF as a standardized instrument for evaluating crisis support implementation in mental health applications and assessing its inter-rater reliability, and characterizing current crisis support quality across six commercial AI-powered products. The sections below address each objective in turn before considering broader implications and limitations.

### 4.1 Reliability of MHACSAF and validity considerations

MHACSAF demonstrated strong inter-rater reliability. The obtained Fleiss’ kappa of 0.87 falls in the ‘almost perfect agreement’ range according to the benchmark scale proposed by Landis and Koch (10), which defines this category as *κ* > 0.80. The ICC(2,1) of 0.94 exceeds the ‘excellent’ threshold (ICC > 0.90) proposed by Koo and Li (8). Both indices confirm H1 and indicate that MHACSAF can yield consistent results when administered by independently trained clinical evaluators working from the same scoring rubric.

These reliability values compare favorably with published data for other app evaluation instruments. Lagan et al. (9) identified over 45 app evaluation frameworks in a systematic scoping review and found that the majority lacked any published reliability data; those that did typically reported moderate agreement in the *κ* = 0.40–0.60 range. Nouri et al. (19) similarly noted that inter-rater reliability is rarely assessed in mHealth evaluation tool development. MHACSAF's reliability profile therefore represents a methodological advance over most existing instruments in this domain.

The reliability observed is attributable in part to the instrument's design. By anchoring each item to discrete, observable interface characteristics – number of navigation steps, presence of tap-to-call functionality, VoiceOver compatibility – MHACSAF reduces the evaluator inference required at each scoring decision. This operationalization strategy is consistent with recommendations from implementation science for developing reproducible fidelity measures that maximize inter-rater agreement by minimizing reliance on subjective judgment (22). The structured four-phase development process (Figure 2), including three rounds of expert review and pilot testing before formal evaluation, further contributed to item clarity.

Content validity rests on the iterative expert consensus process documented in Section 2, which grounded item selection in the three theoretical bodies illustrated in Figure 1: WHO crisis line guidance (34), the Safety Planning Intervention model (26, 27), and established digital health evaluation principles (6, 9, 31). Formal content validity indices were not computed, representing a limitation to be addressed in future work. Construct validity also remains to be established. Convergent validity – assessed by correlating MHACSAF scores with ratings from established instruments such as the American Psychiatric Association's App Evaluation Model (1) or the NICE evidence standards framework (18) – constitutes a priority for future research. Such comparisons would clarify the extent to which crisis support quality, as measured by MHACSAF, is correlated with or independent from broader app quality dimensions such as evidence base, privacy, and usability.

Ecological validity is a further consideration. Expert evaluators working from standardized prompts may not fully replicate the experience of a user in acute crisis, who may be cognitively constricted (24), distressed, or unfamiliar with the application. Incorporating evaluation data from individuals with lived experience of mental health crises – a user-centered validation approach – would complement expert assessment and capture usability dimensions that clinicians may systematically underestimate (30). Future validation studies should embed such participatory methods alongside expert rating.

### 4.2 Current state of crisis support in AI mental health applications

In direct response to RQ1: current commercial AI-powered mental health applications implement crisis support features at levels substantially below established standards across all seven MHACSAF dimensions. No application achieved a ‘Good’ or ‘Excellent’ rating; mean total scores clustered in the ‘Poor’ range (M = 24.9, SD = 9.3 out of 65), and even the highest-performing application, Wysa (M = 40.3), reached only the upper end of the ‘Adequate’ category. These findings are consistent with and extend prior work. Parrish et al. (20) found that among 116 commercially available mental health apps, fewer than half provided any form of crisis resource, and those that did rarely followed evidence-based safety planning protocols. Larsen et al. (11) documented widespread inconsistency in the implementation of safety-relevant features across smartphone tools for suicide prevention. Martinengo et al. (16) similarly reported that most depression and suicide prevention apps lacked functional safety planning components and adequate risk assessment features. The present study extends this literature to the current generation of AI-driven conversational products, demonstrating that the addition of large language model capabilities has not resolved the underlying crisis support deficiencies these earlier reviews identified.

More recent evidence confirms that these deficiencies remain ongoing. Dwyer et al. (2) assessed crisis resources across a sample of 302 commercially available mental health apps and found that only 15% incorporated the 988 Suicide and Crisis Lifeline – the US national standard crisis hotline – and that 14 applications with over 3.5 million combined downloads provided incorrect or non-functional crisis hotlines. The present study extends this literature by demonstrating, through systematic item-level scoring across seven dimensions, how these deficiencies manifest in specific design failures: buried navigation, non-functional links, missing offline functionality, and absent accessibility accommodations. The convergence between MHACSAF pilot findings and the broader app landscape surveyed by Dwyer et al. (2) suggests that the crisis support inadequacies observed here are not idiosyncratic to the six evaluated products but reflect a field-wide pattern.

Technical accessibility failure was among the most clinically significant findings. As shown in Figure 3, Technical Accessibility was among the weakest dimensions across virtually all applications, alongside Awareness and Onboarding (each averaging approximately 17% of the maximum possible score). Three applications (Ash, Earkick, Youper) scored exactly 0; only Wysa, Flourish, and Replika achieved any non-zero score, and even among these, Flourish's mean of 0.3 reflects marginal performance from a single rater rather than consistent functional support. This is not a peripheral concern: mental health conditions co-occur with visual, motor, and cognitive disabilities at substantially elevated rates compared with the general population (21, 32). An application that cannot be navigated by a screen-reader user offers no safety benefit to that population regardless of how well-designed its crisis content is. The near-universal accessibility failure in the evaluated products indicates that accessibility has not been integrated into the design or quality assurance processes of these applications – a pattern also documented for mental health apps more broadly (5).

The pattern of outsourcing crisis information to external platforms represents a systemic safety risk that has not been previously quantified at the item level. Youper directed users to non-functional bitly links and a Wikipedia page; Ash's approach via Find a Helpline, while yielding better-quality information, introduced dependency on a third-party service subject to downtime. Evidence on crisis service effectiveness consistently emphasizes that minimizing barriers between distress recognition and help-seeking is a necessary condition for intervention impact (4, 15, 34). Both approaches fail to guarantee that a user who has located a crisis resource can successfully use it.

The universal reliance on conversational AI as the primary gateway to crisis resources raises particular concerns. All six applications required users to articulate distress through natural language dialogue before crisis resources were surfaced. Stanley and Brown (26) and Stanley et al. (27) demonstrate that safety planning is most effective when delivered promptly and with minimal friction; requiring users in crisis to initiate and sustain multi-turn conversations before receiving support introduces precisely the friction that safety planning models are designed to eliminate. Recent evaluation evidence reinforces this concern. Sobowale et al. (25) evaluated five generative AI chatbots widely used by youth and found that while chatbots performed adequately on accessibility, they performed poorly on risk monitoring and assessment, with raters characterizing crisis handling as qualitatively inadequate; the authors concluded that direct-to-consumer AI chatbots pose unacceptable safety risks for users seeking mental health support. Li et al. (13), in a systematic review and meta-analysis of AI-based conversational agents for mental health, noted that evidence for their efficacy was preliminary and that safety evaluation – including crisis response capability – remained a critical gap in the literature. Multiple access modalities – direct menu access, persistent footer buttons, and proactive detection – should complement rather than substitute for conversational pathways.

No application provided the personal safety contact functionality that is central to evidence-based safety planning (26). This gap is particularly difficult to justify on technical grounds, given that contact storage and one-tap calling are standard smartphone platform capabilities. Its persistence across six products from different developers suggests that the gap reflects a shared design philosophy – in which crisis support is positioned as a reactive add-on rather than a core product feature – rather than a technical constraint. Earkick's explicit disclaimers regarding crisis responsibility illustrate the extreme end of this philosophy.

### 4.3 Implications for practice and future research

For developers, the dimensional findings identify concrete improvement targets. Crisis resources should be reachable within one or two interactions from any screen; offline caching of essential content (hotline numbers, safety plan templates, emergency contacts) is technically straightforward and would address the universal offline functionality gap. Personal safety contact features require only standard platform APIs. Accessibility compliance with WCAG 2.2 and ARIA standards is a legal requirement in many jurisdictions and a practical necessity for reaching the full target population.

For researchers, MHACSAF provides a reproducible instrument for benchmarking products, tracking implementation changes over time, and communicating crisis support quality to stakeholders in a standardized format. It can serve as a component of broader evaluation approaches. MHACSAF could complement instruments such as the APA App Evaluation Model (1) – which addresses evidence base, privacy, and clinical foundation alongside safety – or the Mobile App Rating Scale (28), which assesses engagement, functionality, and information quality. Using MHACSAF alongside these instruments would provide a more complete picture of an application's overall quality profile. Future studies should examine associations between MHACSAF scores and clinical outcomes: do users of higher-scoring apps have better crisis engagement, lower distress following in-app crisis encounters, or higher rates of help-seeking? Linardon et al. (14), in a meta-analysis of 176 randomized controlled trials of mental health smartphone apps, found evidence for efficacy on depressive and anxiety symptoms, but noted that safety outcomes and crisis response were not assessed in most trials – precisely the gap that outcome studies using MHACSAF could address. Torous et al. (29), reviewing current evidence and implementation challenges for smartphone apps, generative AI, and virtual reality in mental health, similarly identified crisis response quality and safety evaluation as under-researched priority areas for the next generation of digital mental health research.

For clinicians recommending mental health apps to patients, the findings suggest that crisis support adequacy should be evaluated explicitly as part of recommendation decisions. Current app recommendation practices frequently rely on general quality indicators (evidence base, user ratings, clinical endorsement) that do not capture crisis support readiness. Given that users may experience acute distress while engaging with these applications, clinicians bear a professional responsibility to assess whether the products they recommend are equipped to respond appropriately.

For regulators and policymakers, MHACSAF offers a structured, reproducible methodology that could inform auditing standards for mental health applications classified as software as a medical device (SaMD). Regulatory frameworks including the FDA's Digital Health Center of Excellence guidance and the EU Medical Device Regulation increasingly require that SaMD developers demonstrate safety for foreseeable use cases; acute psychological distress is among the most foreseeable use cases for any mental health application. A standardized instrument such as MHACSAF could support pre-market review, post-market surveillance, and mandatory minimum standards for crisis support implementation across app marketplaces.

### 4.4 Limitations

This study has several limitations. First, the sample of six applications was selected to represent the range of AI-powered mental health products rather than to constitute a representative probability sample; findings may not generalize to all products in this category. Second, assessment was conducted on iOS only; Android implementations may differ substantially in navigation architecture, platform-specific accessibility features, and feature availability due to version-lagged rollouts. Android and iOS differ in several dimensions directly relevant to MHACSAF criteria: Android's TalkBack screen reader and accessibility API differ from iOS VoiceOver in ways that affect technical accessibility scores; Google Play Store content policies regarding crisis and self-harm content have historically diverged from Apple App Store guidelines, potentially influencing how apps implement safety features on each platform; and permission models for device contact access – relevant to personal safety contact functionality – differ between operating systems. Future studies should assess both platforms in parallel to enable direct comparison and establish whether crisis support deficiencies are platform-specific or systemic. Third, evaluation occurred at a single point in time; applications update frequently and the crisis support features of current versions may differ from those assessed here. Fourth, MHACSAF captures implementation as visible to external evaluators; it does not assess backend functionality such as whether keyword detection triggers human review, nor does it capture actual user experiences during crises. Fifth, the framework does not verify the accuracy or currency of crisis resource information – an important dimension that requires different assessment methods. Sixth, the evaluation panel consisted of English-speaking professionals in specific geographic contexts; assessments of localization and language support may not fully reflect the experiences of users in other regions or language communities. Seventh, content validity indices and convergent validity data were not obtained in this pilot study; these represent priorities for future validation work. Eighth, statistical analyses were conducted by the authors without independent review by a biostatistician. Although the analytic approach is standard and well-documented (Fleiss’ kappa, ICC(2,1), bootstrap confidence intervals), independent statistical review is recommended before the instrument is applied in larger-scale studies.

Taken together, the findings reported here address both objectives of the study. With respect to the first objective, MHACSAF demonstrated the reliability and internal consistency properties necessary for a reproducible research instrument, confirming H1 and establishing a foundation for subsequent validity work. With respect to the second objective, the pilot evaluation produced the first systematic, multi-dimensional characterization of crisis support quality in commercial AI mental health applications, revealing a consistent and clinically significant pattern of inadequacy that persists across products, developers, and crisis support domains. The convergence of these two contributions – a validated instrument and an empirical baseline – positions MHACSAF as a practical tool for measuring progress as the field works toward the safety standard that users in crisis deserve.

## 5 Conclusion

This study introduced MHACSAF, a theoretically grounded instrument for evaluating crisis support implementation in mental health applications, and demonstrated its reliability through independent pilot application to six commercial AI-powered products. The instrument's strong inter-rater agreement (Fleiss’ kappa = 0.87; ICC(2,1) = 0.94) establishes it as a reproducible measurement tool. The pilot findings document a consistent and clinically significant pattern of inadequacy: no evaluated application implemented crisis support at a level meeting established standards, and several introduced active barriers to help-seeking through broken links, non-functional hotlines, and near-total absence of accessibility accommodations.

The broader significance of these findings lies in what they reveal about the field as a whole. The evaluated applications collectively represent millions of users who may turn to them during acute distress. The gap between what these products offer and what evidence-based crisis support requires is not a gap in technical capability – it is a gap in design priority. MHACSAF makes that gap measurable. With a validated instrument now available, researchers, developers, regulators, and clinicians can move from documenting inadequacy to systematically tracking and demanding improvement. Ensuring that mental health applications are safe for users in crisis is not a peripheral quality concern: it is the foundational condition on which any other benefit these products may offer depends.

## Data Availability

The original contributions presented in the study are included in the article/Supplementary Material, further inquiries can be directed to the corresponding author.

## References

[1] American Psychiatric Association. The App Evaluation Model. Washington, DC: APA (2019).

[2] Dwyer B Mikkelson J Burns J Diaz-Pacheco V Torous J. Mental health apps and crisis support: exploring the impact of 988. Psychiatr Serv. (2025) 76(10):867–71. 10.1176/appi.ps.20240485 40836663

[3] Firth J Torous J Nicholas J Carney R Pratap A Rosenbaum S. The efficacy of smartphone-based mental health interventions for depressive symptoms: a meta-analysis of randomized controlled trials. World Psychiatry. (2017) 16(3):287–98. 10.1002/wps.20472 28941113 PMC5608852

[4] Gould MS Kalafat J Harris Munfakh JL Kleinman M. An evaluation of crisis hotline outcomes part 2: suicidal callers. Suicide Life Threat Behav. (2007) 37(3):338–52. 10.1521/suli.2007.37.3.338 17579545

[5] Huckvale K Torous J Larsen ME. Assessment of the data sharing and privacy practices of smartphone apps for depression and smoking cessation. JAMA Netw. Open. (2019) 2(4):e192542. 10.1001/jamanetworkopen.2019.2542 31002321 PMC6481440

[6] International Organization for Standardization. ISO 9241-11:2018 Ergonomics of Human-system interaction – Part 11: Usability: Definitions and Concepts. Geneva: ISO (2018).

[7] Joiner T. Why People Die by Suicide. Cambridge, MA: Harvard University Press (2005).

[8] Koo TK Li MY. A guideline of selecting and reporting intraclass correlation coefficients for reliability research. J Chiropr Med. (2016) 15(2):155–63. 10.1016/j.jcm.2016.02.012 27330520 PMC4913118

[9] Lagan S Sandler L Torous J. Evaluating evaluation frameworks: a scoping review of frameworks for assessing health apps. BMJ Open. (2021) 11:e047001. 10.1136/bmjopen-2020-047001 33741674 PMC7986656

[10] Landis JR Koch GG. The measurement of observer agreement for categorical data. Biometrics. (1977) 33(1):159–74. 10.2307/2529310 843571

[11] Larsen ME Nicholas J Christensen H. A systematic assessment of smartphone tools for suicide prevention. PLoS One. (2016) 11(4):e0152285. 10.1371/journal.pone.0152285 27073900 PMC4830444

[12] Lattie EG Adkins EC Winquist N Stiles-Shields C Wafford QE Graham AK. Digital mental health interventions for depression, anxiety, and enhancement of psychological well-being among college students: systematic review. JMIR Ment Health. (2019) 6(7):e12869. 10.2196/12869 PMC6681642 31333198

[13] Li H Zhang R Lee Y-C Kraut RE Mohr DC. Systematic review and meta-analysis of AI-based conversational agents for promoting mental health and well-being. NPJ Digit Med. (2023) 6:236. 10.1038/s41746-023-00979-5 38114588 PMC10730549

[14] Linardon J Torous J Firth J Cuijpers P Messer M Fuller-Tyszkiewicz M. Current evidence on the efficacy of mental health smartphone apps for symptoms of depression and anxiety: a meta-analysis of 176 randomized controlled trials. World Psychiatry. (2024) 23(1):139–49. 10.1002/wps.21183 38214614 PMC10785982

[15] Mann JJ Apter A Bertolote J Beautrais A Currier D Haas A. Suicide prevention strategies: a systematic review. JAMA. (2005) 294(16):2064–74. 10.1001/jama.294.16.2064 16249421

[16] Martinengo L Van Galen L Lum E Kowalski M Subramaniam M Car J. Suicide prevention and depression apps’ suicide risk assessment and management: a systematic assessment of adherence to clinical guidelines. BMC Med. (2019) 17 (1): 231. 10.1186/s12916-019-1461-z 31852455 PMC6921471

[17] Mishara BL Daigle MS. Effects of different telephone intervention styles with suicidal callers at two suicide prevention centers: an empirical investigation. Am J Community Psychol. (1997) 25(6):861–85. 10.1023/a:1022269314076 9534222

[18] National Institute for Health and Care Excellence. Evidence Standards Framework for Digital Health Technologies. London: NICE (2019).

[19] Nouri R Kalhori SRN Ghazisaeedi M Marchand G Yasini M. Criteria for assessing the quality of mHealth apps: a systematic review. J Am Med Inform Assoc. (2018) 25(8):1089–98. 10.1093/jamia/ocy050 29788283 PMC7646896

[20] Parrish EM Filip TF Torous J Nebeker C Moore RC Depp CA. Are mental health apps adequately equipped to handle users in crisis? Crisis. (2022) 43(4):289–98. 10.1027/0227-5910/a000785 34042465 PMC8641126

[21] Prince M Patel V Saxena S Maj M Maselko J Phillips MR. No health without mental health. Lancet. (2007) 370(9590):859–77. 10.1016/S0140-6736(07)61238-0 17804063

[22] Proctor E Silmere H Raghavan R Hovmand P Aarons G Bunger A. Outcomes for implementation research: conceptual distinctions, measurement challenges, and research agenda. Adm Policy Ment Health. (2011) 38(2):65–76. 10.1007/s10488-010-0319-7 20957426 PMC3068522

[23] R Core Team. R: A language and environment for statistical computing (version 4.3.2). R Foundation for Statistical Computing (2023). Available online at: https://www.R-project.org/ (Accessed February 15, 2026).

[24] Shneidman ES. Suicide as Psychache: A Clinical Approach to Self-Destructive Behavior. Northvale, NJ: Jason Aronson (1993).

[25] Sobowale K Humphrey DK Zhao SY. Evaluating generative AI psychotherapy chatbots used by youth: cross-sectional study. JMIR Ment Health. (2025) 12:e79838. 10.2196/79838 41370787 PMC12694945

[26] Stanley B Brown GK. Safety planning intervention: a brief intervention to mitigate suicide risk. Cogn Behav Pract. (2012) 19(2):256–64. 10.1016/j.cbpra.2011.01.001

[27] Stanley B Brown GK Brenner LA Galfalvy HC Currier GW Knox KL. Comparison of the safety planning intervention with follow-up vs usual care of suicidal patients treated in the emergency department. JAMA Psychiatry. (2018) 75(9):894–900. 10.1001/jamapsychiatry.2018.1776 29998307 PMC6142908

[28] Stoyanov SR Hides L Kavanagh DJ Zelenko O Tjondronegoro D Mani N. Mobile app rating scale: a new tool for assessing the quality of health mobile apps. JMIR Mhealth Uhealth. (2015) 3(1):e27. 10.2196/mhealth.3422 25760773 PMC4376132

[29] Torous J Linardon J Goldberg SB Sun S Bell I Nicholas J. The evolving field of digital mental health: current evidence and implementation issues for smartphone apps, generative artificial intelligence, and virtual reality. World Psychiatry. (2025) 24(2):156–74. 10.1002/wps.21299 40371757 PMC12079407

[30] Torous J Wisniewski H Bird B Carpenter E David G Eber E. Creating a digital health smartphone app and digital phenotyping platform for mental health and diverse healthcare needs: an interdisciplinary and collaborative approach. J Technol Behav Sci. (2019) 4:73–85. 10.1007/s41347-019-00095-w

[31] World Wide Web Consortium (W3C). Web Content Accessibility Guidelines (WCAG) 2.2. W3C Recommendation (2023).

[32] World Health Organization. World Report on Disability. Geneva: WHO (2011).

[33] World Health Organization. Preventing Suicide: A Global Imperative. Geneva: WHO (2014).

[34] World Health Organization. Preventing Suicide: A Resource for Establishing a Crisis Line. Geneva: WHO (2018).

[35] World Health Organization. LIVE LIFE: An Implementation Guide for Suicide Prevention in Countries. Geneva: WHO (2021).

[36] Zalsman G Hawton K Wasserman D van Heeringen K Arensman E Sarchiapone M. Suicide prevention strategies revisited: 10-year systematic review. Lancet Psychiatry. (2016) 3(7):646–59. 10.1016/S2215-0366(16)30030-X 27289303

