# Supplementary material for: Mental health app crisis support assessment framework: development and pilot testing
Source: Front Digit Health. 2026 Jun 10;8:1814547. doi: 10.3389/fdgth.2026.1814547 (PMC13290974; doi:10.3389/fdgth.2026.1814547)
Supplement: Supplementary file 1 [file Datasheet1.docx]

Supplementary Material

# Supplementary Material S1: MHACSAF Scoring Rubric

This rubric provides item-level scoring anchors for all sections of the Mental Health App Crisis Support Assessment Framework (MHACSAF). Each item specifies the criterion being evaluated, the maximum points available, and concrete behavioral anchors for zero, partial, and maximum scores. Evaluators should score each item independently and record a written justification for each scoring decision.

General instructions: evaluate each item based on observable interface features accessible to a user within the app’s standard navigation. Do not infer functionality that is not directly demonstrable. When an item falls between anchors, award the lower score unless the unmet criterion is clearly inconsequential to crisis support functionality.

# Supplementary Tables

**Supplementary Table S1.1. Section 0. Eligibility Screening**

*This section determines whether the application is eligible for scored assessment. Applications scoring 0 on Item 0.1 are classified as ‘Not Eligible’ and receive a total score of 0. Do not proceed to Sections 1–7.*

| **Item** | **Criterion** | **Score** | **Anchor – Score 0** | **Anchor – Score 1** |
| --- | --- | --- | --- | --- |
| 0.1 | Does the application contain any content or functionality designated for crisis or acute distress situations (e.g., safety resources, crisis lines, mental health emergency information)? | 0 or 1 | No crisis-specific content or functionality is present. General wellness content does not qualify. | At least one element specifically addressing crisis or acute distress is present and accessible within the app. |

**Supplementary Table S1.2. Section 1. Ease of Access (12 points)**

*Score each indicator independently. For Item 1.1, record the exact number of taps from the home screen to the primary crisis resource. For Item 1.4, disable Wi-Fi and mobile data before testing.*

| **Item** | **Criterion** | **Max pts** | **Anchor – 0 pts** | **Anchor – 1 pt** | **Anchor – max pts** |
| --- | --- | --- | --- | --- | --- |
| 1.1 | Navigation depth: minimum number of interactions required to reach primary crisis resources from the home screen. | 4 | Crisis resources require 5 or more interactions or cannot be found through navigation (search only). | Crisis resources require 3–4 interactions. | Crisis resources require 1–2 interactions. Direct shortcut from home screen (e.g., persistent button, top-level menu item). Award 4 pts. |
| 1.2 | Multiple access pathways: crisis resources can be reached via at least two different navigation routes (e.g., menu + chat + search). | 2 | Only one route to crisis resources exists. | Two independent routes exist. | Three or more independent routes exist. Award 2 pts. |
| 1.3 | Proactive crisis detection: the app identifies expressions of acute distress or crisis and proactively surfaces crisis resources without user navigating to them explicitly. | 3 | No proactive detection. Crisis resources surface only through deliberate user navigation. | App detects crisis expressions but requires multiple conversational turns before surfacing resources (≥3 turns). | App detects crisis expressions and surfaces resources within 1–2 turns. Direct link or action is provided. Award 3 pts. |
| 1.4 | Offline availability: primary crisis resources (hotline numbers, safety plan, emergency contacts) remain accessible without an active internet connection. | 3 | Crisis resources are entirely unavailable offline. | Some crisis content is available offline but core resources (hotlines, emergency contacts) are not. | Core crisis resources are accessible offline without degradation. Award 3 pts. |

**Supplementary Table S1.3. Section 2. Coverage and Prioritization (24 points)**

*For Items 2.1–2.5, use the standardized crisis prompts specified in the evaluation protocol. A crisis presentation is considered ‘covered’ only when a dedicated, observable response or resource is provided – not when the app simply continues a generic chat conversation.*

| **Item** | **Criterion** | **Max pts** | **Anchor – 0 pts** | **Anchor – partial** | **Anchor – max pts** |
| --- | --- | --- | --- | --- | --- |
| 2.1 | Coverage of suicidal ideation or intent: app provides dedicated support (not merely a chat response) for users expressing suicidal ideation. | 2 | No dedicated support. Only a generic chat response or no response. | Detects expression and refers to external resource only, without in-app support. | Provides in-app response plus at least one active resource (hotline, safety plan, or crisis text line). Award 2 pts. |
| 2.2 | Coverage of panic attacks: app provides dedicated support for users experiencing panic attacks. | 2 | No dedicated support. | Partial: identifies state but offers only generic coping advice without crisis-specific resources. | Provides targeted guidance (e.g., grounding, breathing) plus referral to appropriate service if needed. Award 2 pts. |
| 2.3 | Coverage of self-harm urges: app provides dedicated support for users reporting urges to engage in non-suicidal self-harm. | 2 | No dedicated support. Topic is ignored or deflected. | Identifies topic but response is limited to a generic wellness message. | Provides targeted support including a coping strategy and referral. Award 2 pts. |
| 2.4 | Coverage of acute anxiety or distress: app provides dedicated support for acute anxiety episodes. | 2 | No dedicated support. | Identifies state but offers only generic encouragement. | Provides targeted coping support and escalation pathway. Award 2 pts. |
| 2.5 | Coverage of substance-related crises: app provides dedicated support for substance use emergencies. | 2 | No dedicated support. Topic absent or ignored. | Identifies topic but provides only a generic response. | Provides substance-specific resource (hotline, guidance) and escalation option. Award 2 pts. |
| 2.6 | Multiple support modalities per crisis state: for at least one crisis presentation, the app offers more than one type of support (e.g., hotline + grounding exercise + chat). | 3 | Only one modality per crisis state across all presentations. | Two modalities for at least one crisis state. | Three or more modalities for at least one crisis state. Award 3 pts. |
| 2.7 | Personal safety contact functionality: users can store personal emergency contacts (name + phone number) and access them quickly during a crisis. | 3 | No personal contact storage. | Names only – no phone numbers stored; no quick-dial. | Phone numbers stored with one-tap call functionality. Award 3 pts. |
| 2.8 | Safety planning tool: app includes a structured safety plan (warning signs, coping strategies, contacts, reasons for living, means restriction) as a dedicated interactive feature. | 3 | No safety plan feature. | Basic safety plan missing 2+ components. | Comprehensive interactive safety plan with all core components. Award 3 pts. |
| 2.9 | Reasons-for-living prompts: app includes a feature to identify and record reasons for living. | 1 | Absent. | – | Present as named feature or exercise. Award 1 pt. |
| 2.10 | Lethal means guidance: app includes information about reducing access to means of self-harm. | 2 | Absent. | Brief mention without actionable guidance. | Dedicated, actionable guidance on means reduction. Award 2 pts. |
| 2.11 | Coping strategies library: app provides a curated collection of coping strategies accessible in or near crisis features. | 2 | No coping library. | Coping content exists but is not connected to or surfaced during crisis interactions. | Coping library directly integrated into crisis support flow. Award 2 pts. |

**Supplementary Table S1.4. Section 3. Hotlines and Emergency Services (13 points)**

*Test all tap-to-call buttons by initiating calls (it is not necessary to complete the call). Record whether each link or button functions as expected. Links that open an external webpage rather than initiating a call do not qualify as tap-to-call.*

| **Item** | **Criterion** | **Max pts** | **Anchor – 0 pts** | **Anchor – partial** | **Anchor – max pts** |
| --- | --- | --- | --- | --- | --- |
| 3.1 | Number of crisis hotlines listed (excluding emergency services). | 3 | Zero hotlines listed. | 1 hotline listed. | 2 or more hotlines listed; includes at least one population-specific line (e.g., LGBTQ+, veterans, youth). Award 3 pts. |
| 3.2 | Emergency services access: app includes access to universal emergency services (e.g., 911, 112, 999). | 2 | No emergency services access. | Emergency number listed but not functional (e.g., not tap-to-call). | Functional tap-to-call access to emergency services integrated. Award 2 pts. |
| 3.3 | One-tap calling: user can initiate a call to a crisis hotline with a single tap. | 3 | No tap-to-call. User must manually dial. | Tap-to-call available but requires confirmation step or is buried. | One-tap calling with no intermediate steps. Award 3 pts. |
| 3.4 | Call prioritization: the most urgent or widely recognized crisis line is visually prominent (e.g., listed first, highlighted). | 2 | No visual prioritization. Lines listed in arbitrary order. | National crisis line present but not visually differentiated. | National/primary crisis line is clearly the most prominent contact. Award 2 pts. |
| 3.5 | Operating hours display: hotline listings include information about hours of availability (24/7, or specific times). | 1 | No operating hours information for any hotline. | – | At least one hotline listing includes operating hours or 24/7 indicator. Award 1 pt. |
| 3.6 | Crisis text/online support: app includes at least one non-phone crisis resource (chat, SMS, online). | 2 | No text/online crisis resource. | Text/online resource listed but not functional or requires external navigation. | Functional text or online crisis resource integrated or clearly linked. Award 2 pts. |

**Supplementary Table S1.5. Section 4. Content Clarity (6 points)**

*Assess content as displayed to a user during a crisis interaction, not marketing or general help content. Typography should be evaluated under normal lighting conditions on a physical device.*

| **Item** | **Criterion** | **Max pts** | **Anchor – 0 pts** | **Anchor – partial** | **Anchor – max pts** |
| --- | --- | --- | --- | --- | --- |
| 4.1 | Language clarity: crisis content uses plain language free of clinical jargon, passive voice, or ambiguous phrasing. | 2 | Content is jargon-heavy, confusing, or ambiguous. | Mostly clear but contains some ambiguous or technical phrasing. | All crisis content is clearly written at a level accessible to a distressed user. Award 2 pts. |
| 4.2 | Empathetic phrasing and tone: crisis content uses non-judgmental, compassionate language that acknowledges the user's distress. | 2 | Tone is clinical, dismissive, or stigmatizing. | Neutral tone; not stigmatizing but not empathetically attuned. | Content is explicitly empathetic, validating, and supportive. Award 2 pts. |
| 4.3 | Visual readability: crisis content uses sufficient font size, contrast, and visual hierarchy for readability under acute stress. | 2 | Poor contrast, small font, or no visual hierarchy. Critical information appears as secondary. | Adequate readability but some elements could obscure urgency (e.g., low-contrast color scheme). | High contrast, clearly readable typography, visual hierarchy that guides attention to critical actions. Award 2 pts. |

**Supplementary Table S1.6. Section 5. Technical Accessibility (3 points)**

*Activate iOS VoiceOver (Settings → Accessibility → VoiceOver) before navigating to crisis screens. For touch target size, use developer tools or measure visually against the 44×44 pt standard. Score 0 if screen reader navigation fails on any crisis-critical screen.*

| **Item** | **Criterion** | **Max pts** | **Anchor – 0 pts** | **Anchor – 1 pt** | **Anchor – max pts** |
| --- | --- | --- | --- | --- | --- |
| 5.1 | Screen reader compatibility: crisis support screens are navigable and fully functional with iOS VoiceOver or Android TalkBack enabled. | 1 | Screen reader navigation fails on crisis screens. Elements unlabelled or inaccessible. | – | All crisis support elements are accessible via screen reader. Award 1 pt. |
| 5.2 | Touch target adequacy: interactive elements within crisis screens meet minimum touch target size (44×44 pt per Apple HIG / WCAG 2.2 AAA). | 1 | Interactive elements are too small for reliable activation by users with motor impairments. | – | All interactive crisis elements meet or exceed minimum touch target size. Award 1 pt. |
| 5.3 | Additional assistive technology support: app provides other accessibility accommodations (e.g., Dynamic Type, high contrast mode, haptic feedback) on crisis screens. | 1 | No additional accommodations. | – | At least one additional accessibility accommodation is present and functional. Award 1 pt. |

**Supplementary Table S1.7. Section 6. Location and Language (4 points)**

*Test geographic adaptation by setting device region to a non-US location (e.g., United Kingdom, Germany) and observing whether crisis resources change. Language testing should use device language settings where possible.*

| **Item** | **Criterion** | **Max pts** | **Anchor – 0 pts** | **Anchor – partial** | **Anchor – max pts** |
| --- | --- | --- | --- | --- | --- |
| 6.1 | Geographic customization: app adapts crisis resources (hotlines, emergency numbers) to the user's detected or selected location. | 2 | No geographic adaptation. US-only or default resources regardless of user location. | Manual location selection available but not auto-detected. | Automatic geographic adaptation with location-appropriate resources. Award 2 pts. |
| 6.2 | Language localization: crisis content is available in languages other than English. | 1 | English only. | – | Crisis content available in at least one language other than English. Award 1 pt. |
| 6.3 | Hotline language disclosure: the app indicates which languages each listed crisis hotline supports. | 1 | No language information provided for any hotline. | – | Language(s) supported by each hotline are explicitly stated. Award 1 pt. |

**Supplementary Table S1.8. Section 7. Awareness and Onboarding (3 points)**

*Create a fresh user account to observe the full onboarding sequence without skipping. For Item 7.3, use the app for a minimum of two sessions before concluding that no reminders are sent.*

| **Item** | **Criterion** | **Max pts** | **Anchor – 0 pts** | **Anchor – 1 pt** | **Anchor – max pts** |
| --- | --- | --- | --- | --- | --- |
| 7.1 | Onboarding crisis education: the app introduces crisis features and their purpose during the initial onboarding sequence. | 1 | Crisis features not mentioned during onboarding. | – | Onboarding explicitly introduces crisis features with instructions on how and when to use them. Award 1 pt. |
| 7.2 | In-app explanations: crisis features include contextual guidance explaining their purpose and how to use them. | 1 | No explanatory content accompanying crisis features. | – | At least one crisis feature includes a clear explanation of its purpose and use. Award 1 pt. |
| 7.3 | Proactive reminders: app sends periodic reminders about the availability of crisis features (not only when crisis is detected). | 1 | No proactive reminders. | – | App sends at least one reminder about crisis feature availability during normal use. Award 1 pt. |

**Supplementary Table S1.9. Score Summary Sheet**

*Complete after scoring all sections. Transfer subtotals to this sheet and calculate the total score.*

| **Dimension** | **Max points** | **Score awarded** |
| --- | --- | --- |
| Section 0. Eligibility Screening | Pass/Fail |  |
| Section 1. Ease of Access | 12 |  |
| Section 2. Coverage and Prioritization | 24 |  |
| Section 3. Hotlines and Emergency Services | 13 |  |
| Section 4. Content Clarity | 6 |  |
| Section 5. Technical Accessibility | 3 |  |
| Section 6. Location and Language | 4 |  |
| Section 7. Awareness and Onboarding | 3 |  |
| **TOTAL** | **65** |  |

**Quality classification:** Excellent (53–65) □ Good (41–52.9) □ Adequate (27–40.9) □ Poor (14–26.9) □ Inadequate (0–13.9) □

**Rater name:** ___________________________ **Date of evaluation:** ___________________________

**Application name & version:** ___________________________ **Platform:** ___________________________
