# Supplementary material for: Mental health app crisis support assessment framework: development and pilot testing
Source: Front Digit Health. 2026 Jun 10;8:1814547. doi: 10.3389/fdgth.2026.1814547 (PMC13290974; doi:10.3389/fdgth.2026.1814547)
Supplement: Supplementary file 2 [file Datasheet2.docx]

Supplementary Material

# Supplementary Material S2: Rater–Application Interaction Examples

This supplement provides illustrative examples of rater interactions with each of the six evaluated applications. Because the original screenshots include third-party copyrighted material, each figure has been replaced with a schematic illustration that reproduces only the structural and behavioural features observed during the standardized evaluation protocol (navigation depth, presence or absence of UI elements, content layout, visual hierarchy, and accessibility affordances). App names, version numbers, and platform are retained because they identify the precise build that was evaluated. Specific phone numbers, URLs, and country names shown inside the illustrations are illustrative placeholders unless they are explicitly referenced in the caption. Captions describe what is illustrated and which MHACSAF item(s) the observation informs, and reference the rubric items by section number (see Supplementary Material S1).

## Supplementary Figures


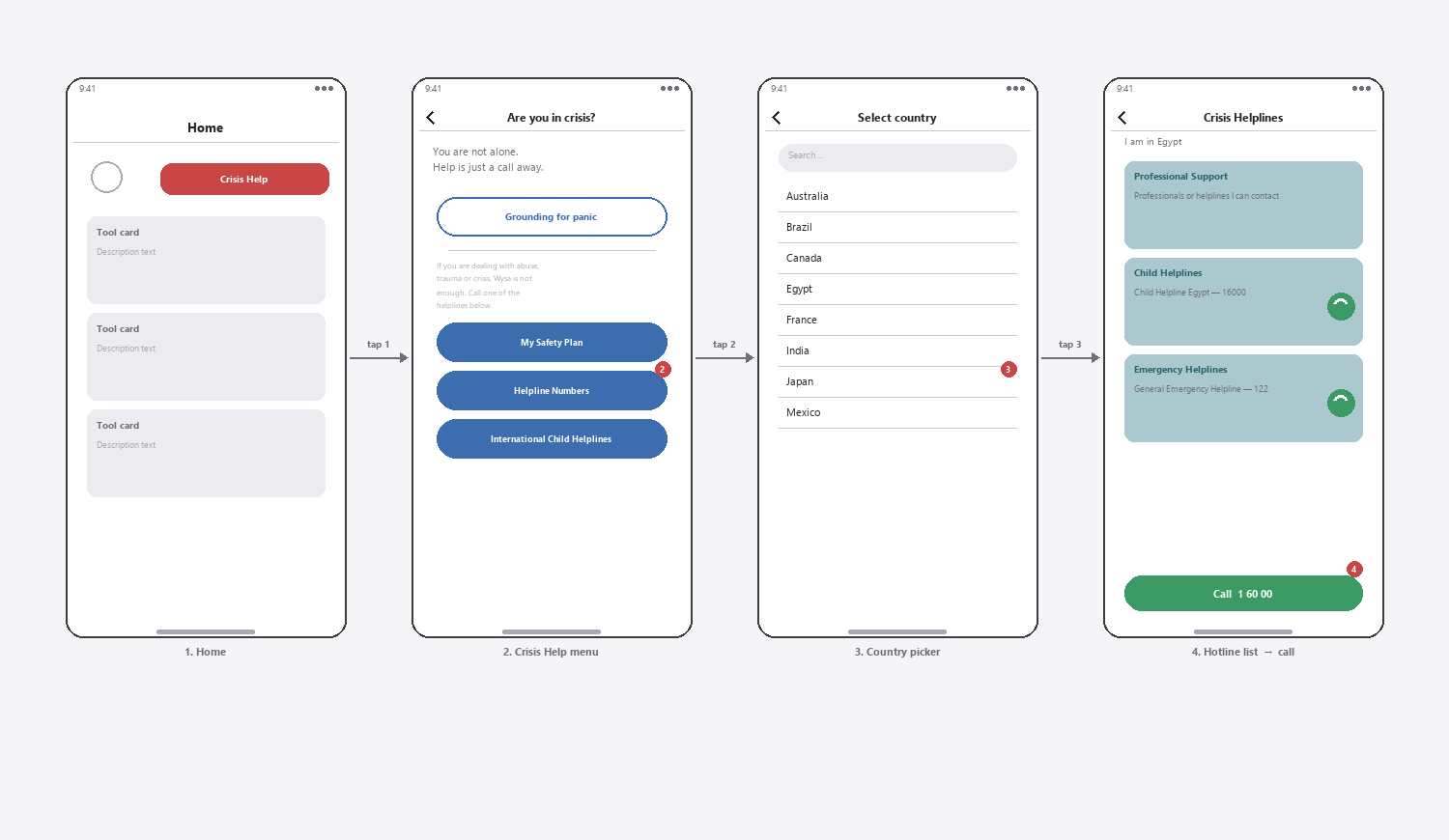


**Supplementary Figure S2.01.** Figure SS25--0011. Wysa (v.6.10.5), iOS. Navigation flow from the home screen to an active crisis hotline call button: Crisis Help menu (tap 1) – category selection (tap 2) – country selection (tap 3) – hotline list (tap 4) – call button. A minimum of four taps separates a user in acute distress from initiating an emergency call, with an additional mandatory country selection step before any hotline number becomes accessible. Illustrates MHACSAF Section 1.1 (navigation depth) and Section 3.3 (absence of one-tap calling).


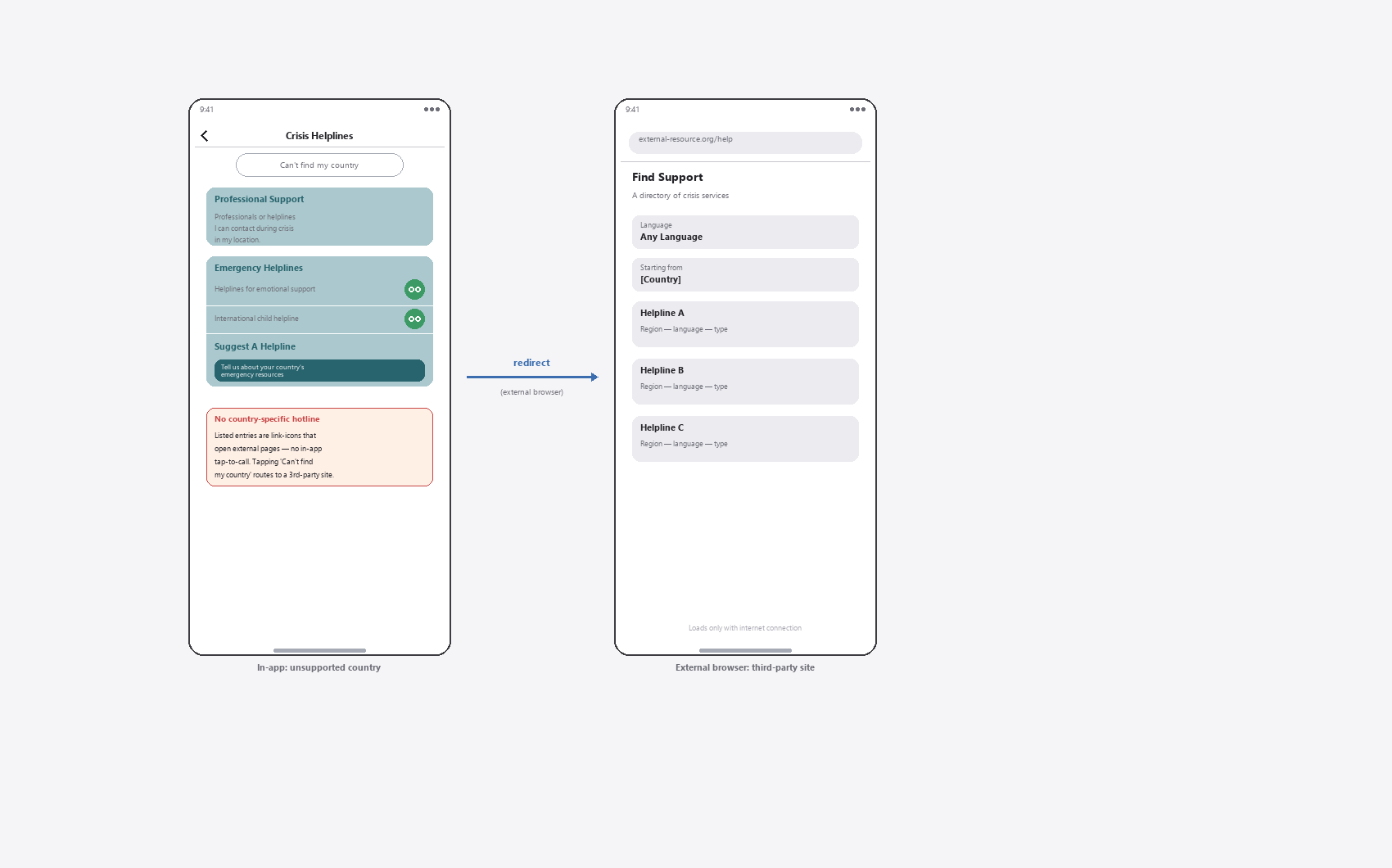


**Supplementary Figure S2.02.** Figure SS25--0022. Wysa (v.6.10.5), iOS. Fallback flow triggered when the user’s country is not listed in the Helpline Numbers database: the app redirects to an external third-party resource rather than providing an in-app callable contact for the user’s country. This pathway is unavailable without an active internet connection, leaving users in unsupported regions without any accessible crisis contact. Illustrates MHACSAF Section 3.1 (hotline coverage gaps) and Section 1.4 (offline availability failure).


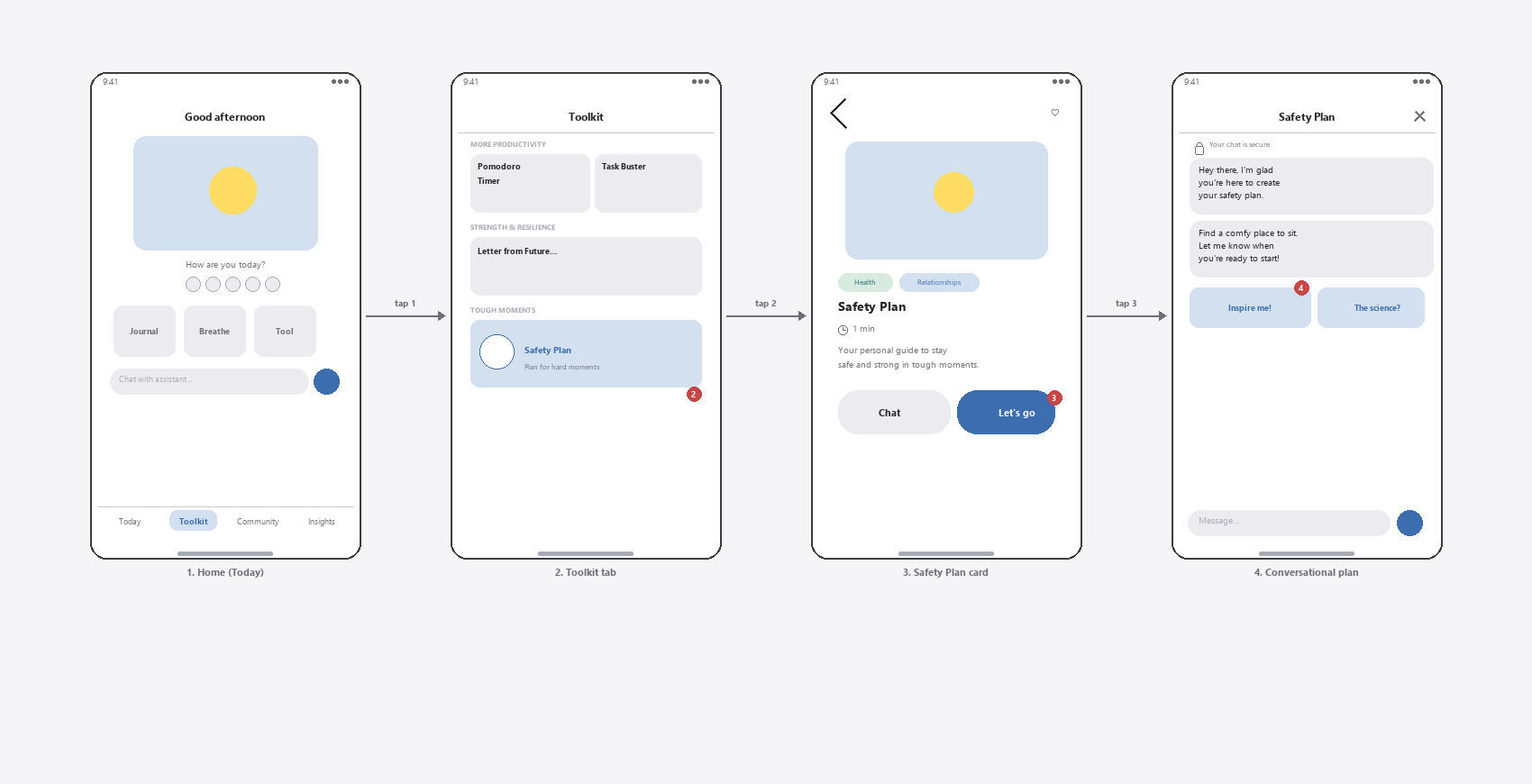


**Supplementary Figure S2.03.** Figure SS25--0033. Flourish (v.2.46.0), iOS. Navigation flow from the home screen to the Safety Plan feature requiring four sequential taps, with no shortcut or persistent crisis button available at any level of the interface. Crisis support in Flourish is organized primarily through conversational chat interactions rather than direct access to emergency resources, meaning a user in acute distress must initiate and sustain a dialogue before any official crisis contact becomes visible. Illustrates MHACSAF Section 1.1 (navigation depth) and Section 3.3 (absence of direct pathway to emergency services).


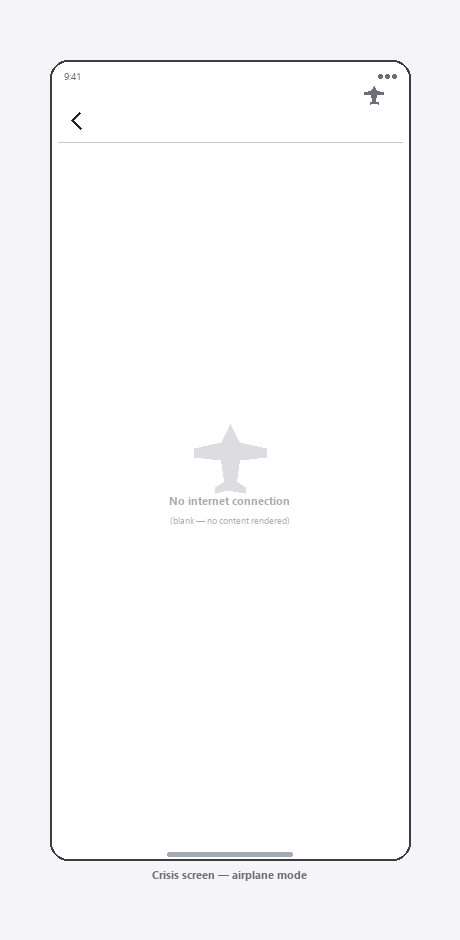


**Supplementary Figure S2.04.** Figure SS25--0044. Wysa (v.6.10.5), iOS, device in Airplane Mode. Blank white screen displayed when attempting to access crisis resources without an active internet connection – no cached content, hotline numbers, or basic safety information is available offline. A user experiencing a mental health crisis in a low-connectivity environment receives no support whatsoever from the application. Illustrates MHACSAF Section 1.4 (offline availability failure).


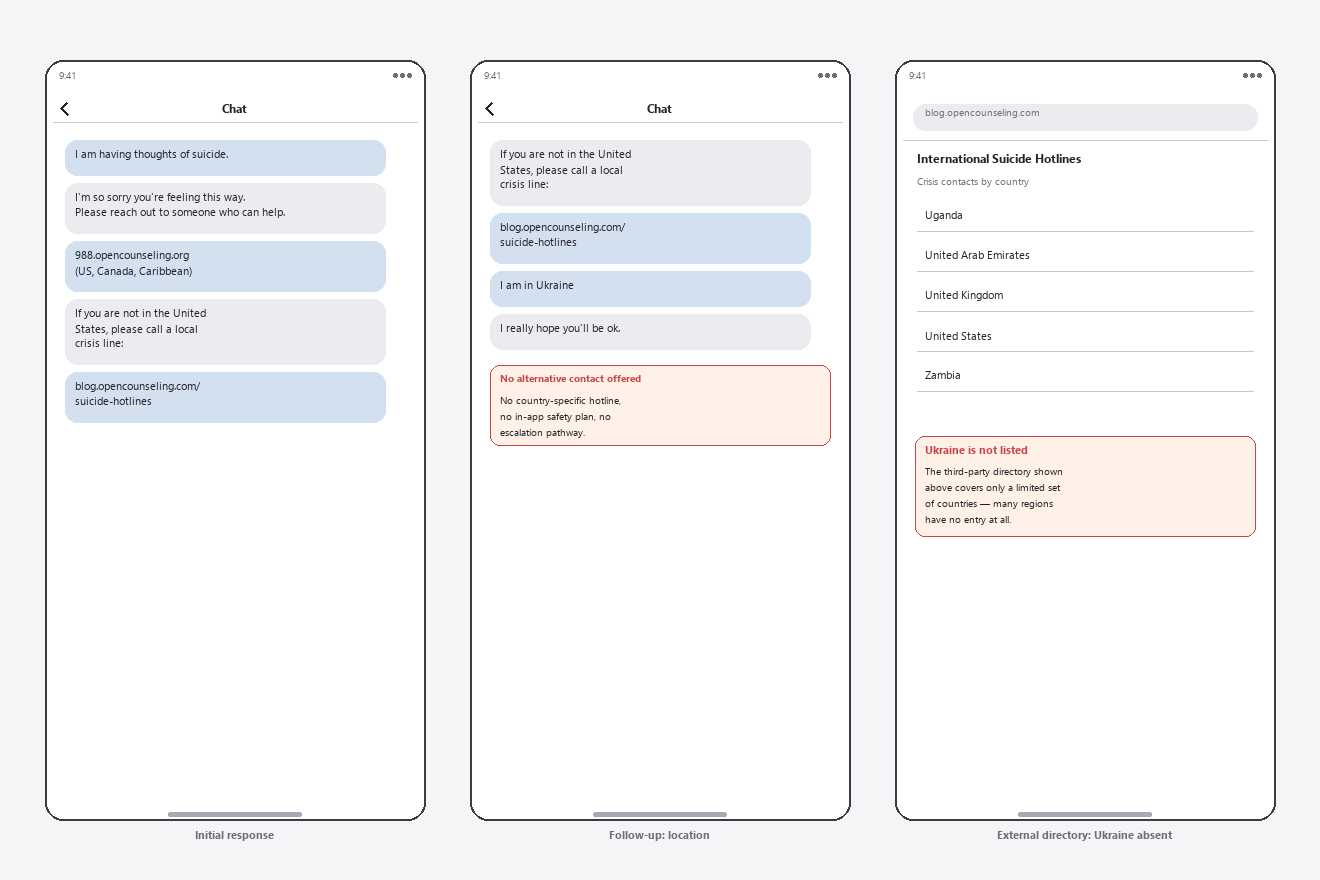


**Supplementary Figure S2.05.** Figure SS25--0055. Replika (v.11.2.1), iOS. Chat sequence in response to a standardized suicidal ideation prompt. The application provides two links: the 988 Suicide and Crisis Lifeline website (US-specific) and blog.opencounseling.com/suicide-hotlines, which lists crisis contacts for a limited number of countries only. When the user follows up with "I am in Ukraine" – a country absent from the provided resources – the application responds with "I really hope you'll be ok" and does not attempt to locate an alternative crisis contact. No in-app safety plan, no local hotline, and no escalation pathway are offered at any point in the interaction. Illustrates MHACSAF Section 2.1 (crisis coverage limited to reactive chat), Section 3.1 (hotline coverage gaps for non-represented countries), and Section 6.1 (absence of geographic adaptation).


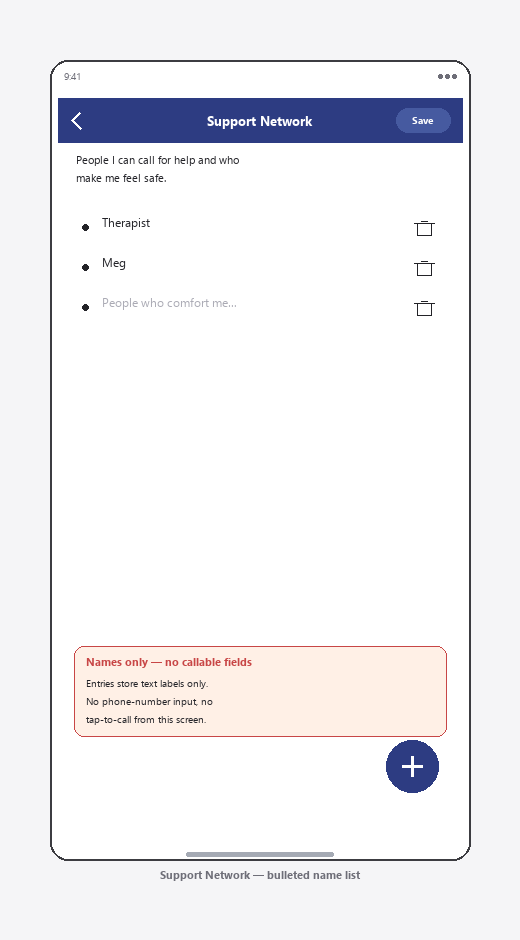


**Supplementary Figure S2.06.** Figure SS25--0066. Wysa (v.6.10.5), iOS. Support Network screen within the Safety Plan feature displaying contact name entries with no option to store phone numbers or initiate a call directly from the interface. A user in crisis who has added trusted contacts to this list cannot reach them through the application – names are recorded but serve no functional emergency purpose. Illustrates MHACSAF Section 2.3 (personal safety contact functionality gap).


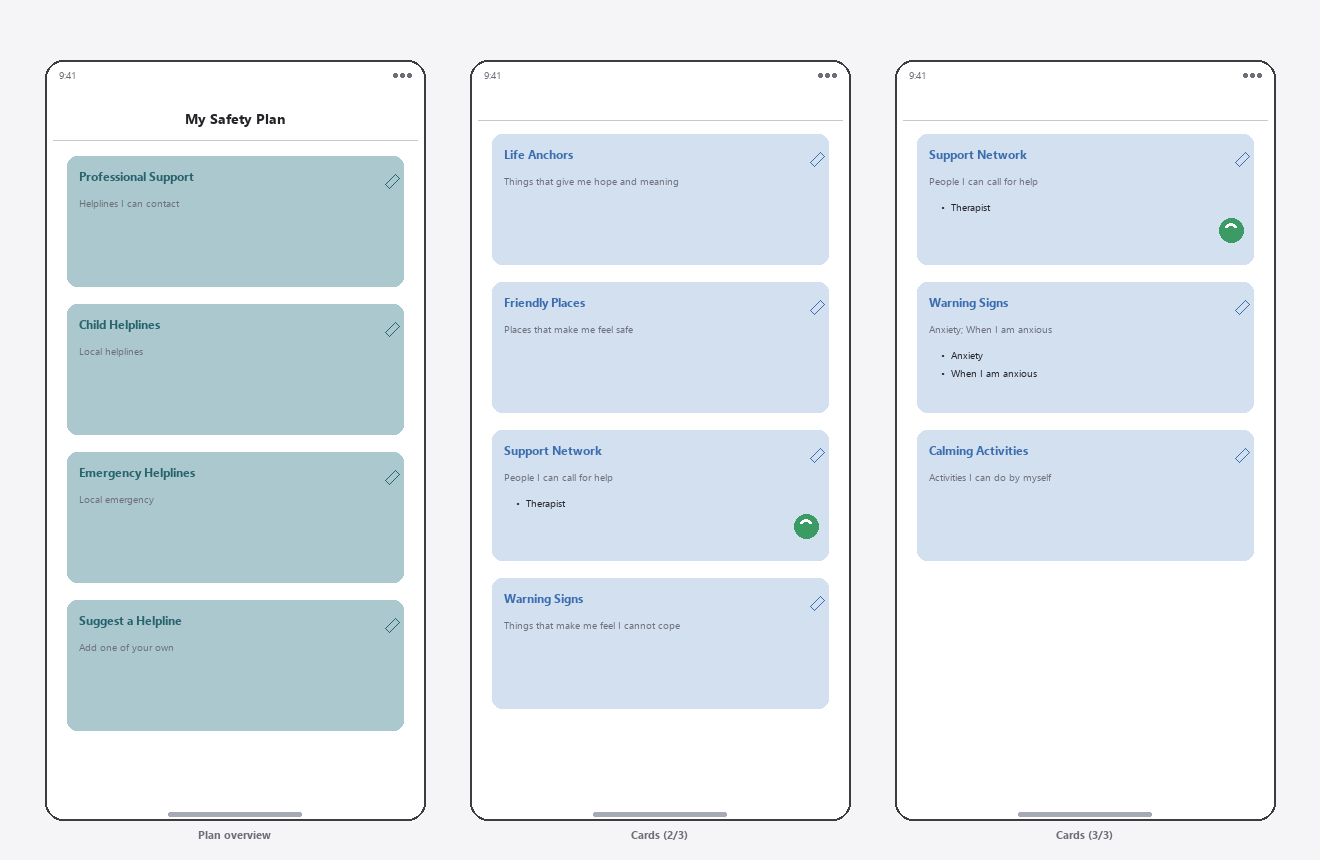


**Supplementary Figure S2.07.** Figure SS25--0077. Wysa (v.6.10.5), iOS. Safety Plan feature displaying all core components of the evidence-based safety planning protocol: personal warning signs, coping activities, support contacts, life anchors, and calming activities. Each component is interactive and saved within the user's profile for repeated access. Illustrates MHACSAF Section 2.5/2.6 (comprehensive safety planning implementation); positive exemplar for the Coverage and Prioritization dimension.


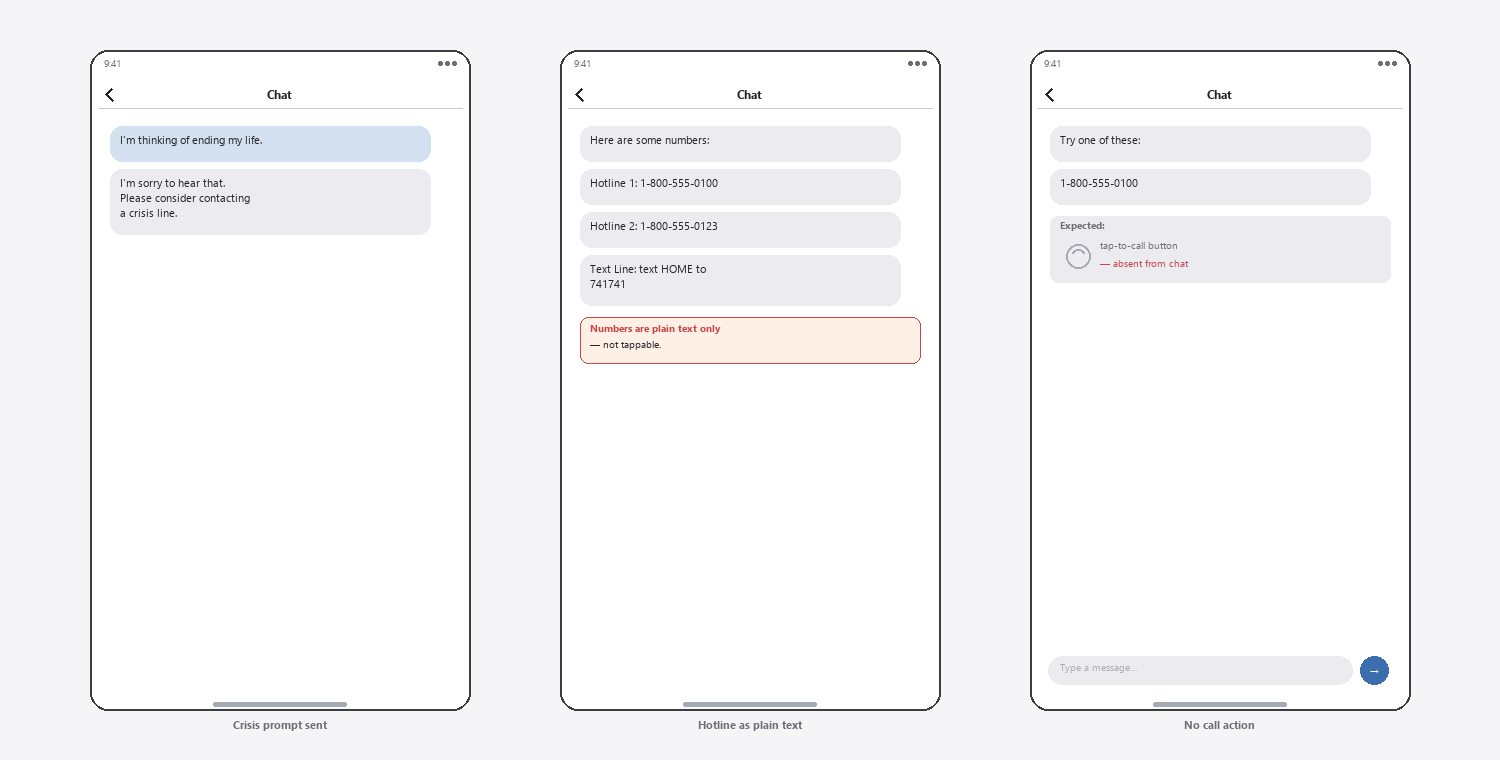


**Supplementary Figure S2.08.** Figure SS25--0088. Earkick (v.2.13.7), iOS. Chat screen showing crisis hotline numbers delivered as plain text within a conversational dialogue, with no tap-to-call functionality, no in-app emergency contact integration, and no alternative crisis resources accessible outside the chat interface. A user must manually dial any number provided, adding friction at the moment of greatest need. Illustrates MHACSAF Section 3.3 (absence of one-tap calling) and Section 3.1 (hotline delivery format inadequate for acute distress).


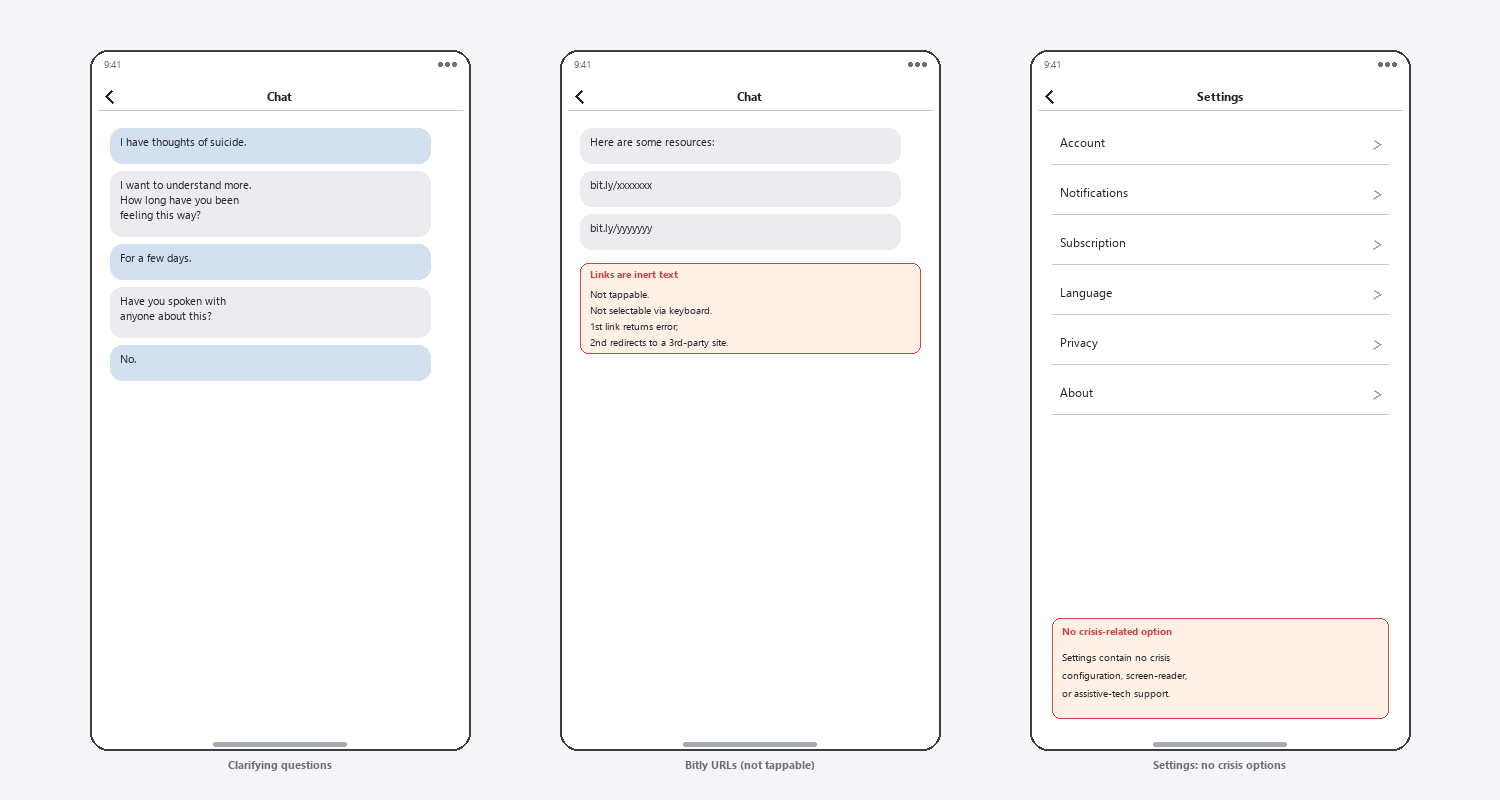


**Supplementary Figure S2.09.** Figure SS25--0099. Youper (v.12.07.003), iOS. Chat sequence following a standardized suicidal ideation prompt: the application asks several clarifying questions before providing two bitly-shortened URLs to external resources. Neither link is tappable nor copyable via the device keyboard, requiring the user to transcribe the URLs manually into a browser. When accessed manually, one link returns an error and fails to load; the second redirects to a third-party website listing crisis hotlines for various countries. No in-app crisis resources, hotline numbers, or safety tools are provided at any point in the interaction. Application settings contain no crisis-related features or configuration options of any kind. The application scored 0 on Technical Accessibility, with no screen-reader support or assistive technology accommodations present on any crisis-relevant screen. Illustrates MHACSAF Section 3.1 (hotline delivery format), Section 3.3 (absence of functional call access), Section 1.1 (multi-turn dialogue required before any resource is surfaced), Section 1.2 (no alternative access pathway outside chat), and Section 5.1 (absence of assistive technology support).


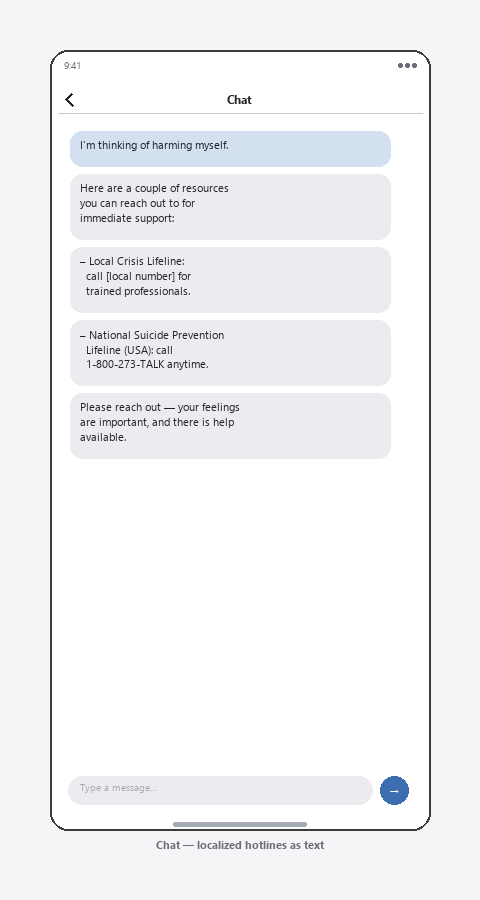


**Supplementary Figure S2.10.** Figure SS25--1100. Earkick (v.2.13.7), iOS. Chat screen showing the application's response to a standardized suicidal ideation prompt: crisis hotline numbers are delivered as plain text within chat messages with no tap-to-call functionality, requiring the user to manually copy or transcribe each number before being able to initiate a call. Each hotline is presented as a separate message with the number rendered as readable plain text, and resources are geographically relevant to the user’s country – demonstrating adequate content localization and information clarity while failing on functional accessibility. A user in acute distress must complete additional manual steps to reach emergency support rather than connecting with a single tap. Illustrates MHACSAF Section 3.3 (absence of one-tap calling), Section 1.1 (multi-step process required to access emergency contact), and Section 6.1 (geographic adaptation present); partial exemplar demonstrating adequate localization alongside a critical usability gap.


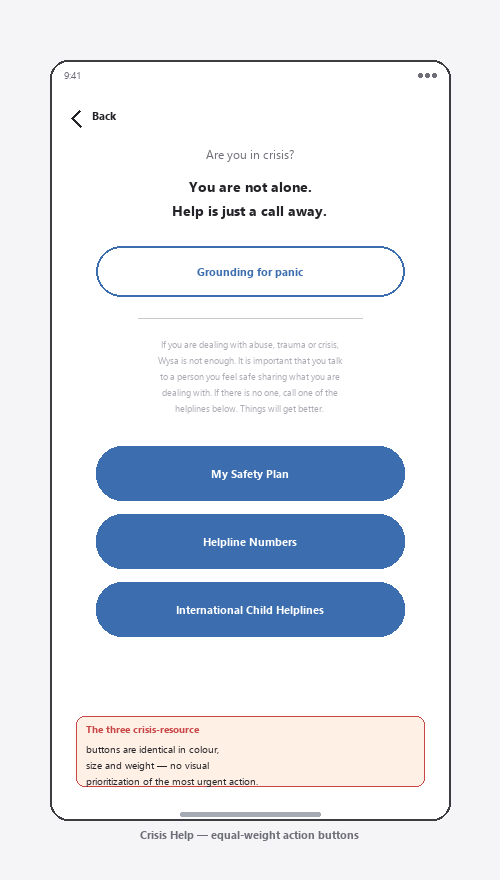


**Supplementary Figure S2.11.** Figure SS25--1111. Wysa (v.6.10.5), iOS. Crisis Help screen with explanatory text rendered in pale grey small-size font that visually recedes against the white background, reducing its perceived importance at the moment it is most critical. The three crisis-resource buttons – My Safety Plan, Helpline Numbers, and International Child Helplines – are presented as a set of visually identical solid blue buttons of equal weight and size, with no logical hierarchy reflecting urgency, user need, or clinical priority. A user in acute distress must read and evaluate all three options equally rather than being guided toward the most appropriate immediate action. Illustrates MHACSAF Section 4.2 (visual clarity failure) and Section 3.4 (absence of visual prioritization of primary crisis contact).


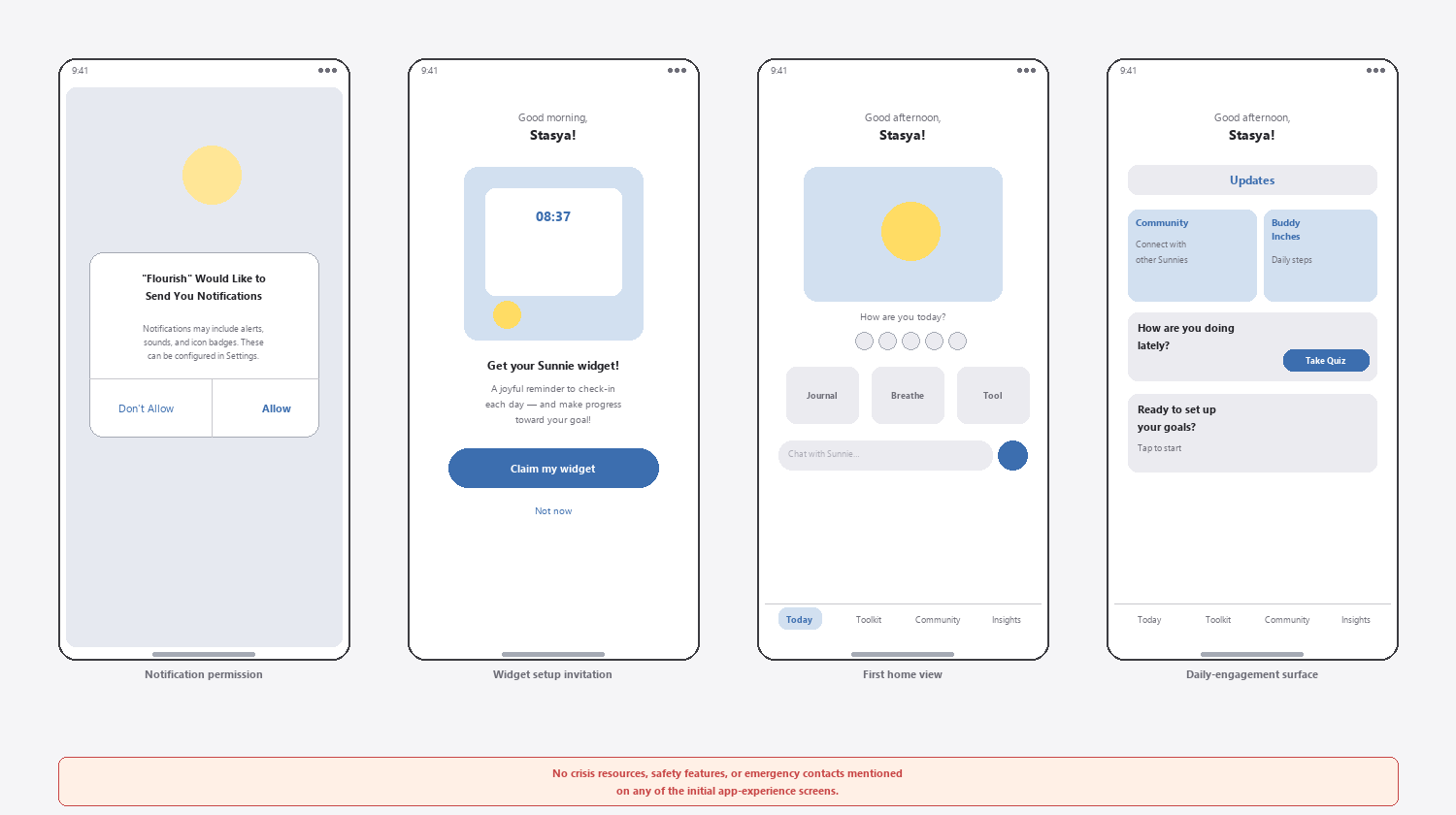


**Supplementary Figure S2.12.** Figure SS25--1122. Flourish (v.2.46.0), iOS. Initial app-experience screens encountered immediately after account creation (notification permission prompt, widget setup invitation, first home view, and early daily-engagement surfaces) with no mention of crisis resources, safety features, or emergency contacts at any stage. A user who installs the application during or preceding a period of acute distress receives no information about available crisis support until they independently discover it through navigation or chat interaction. Illustrates MHACSAF Section 7.1 (absence of crisis feature education during onboarding and initial app experience).


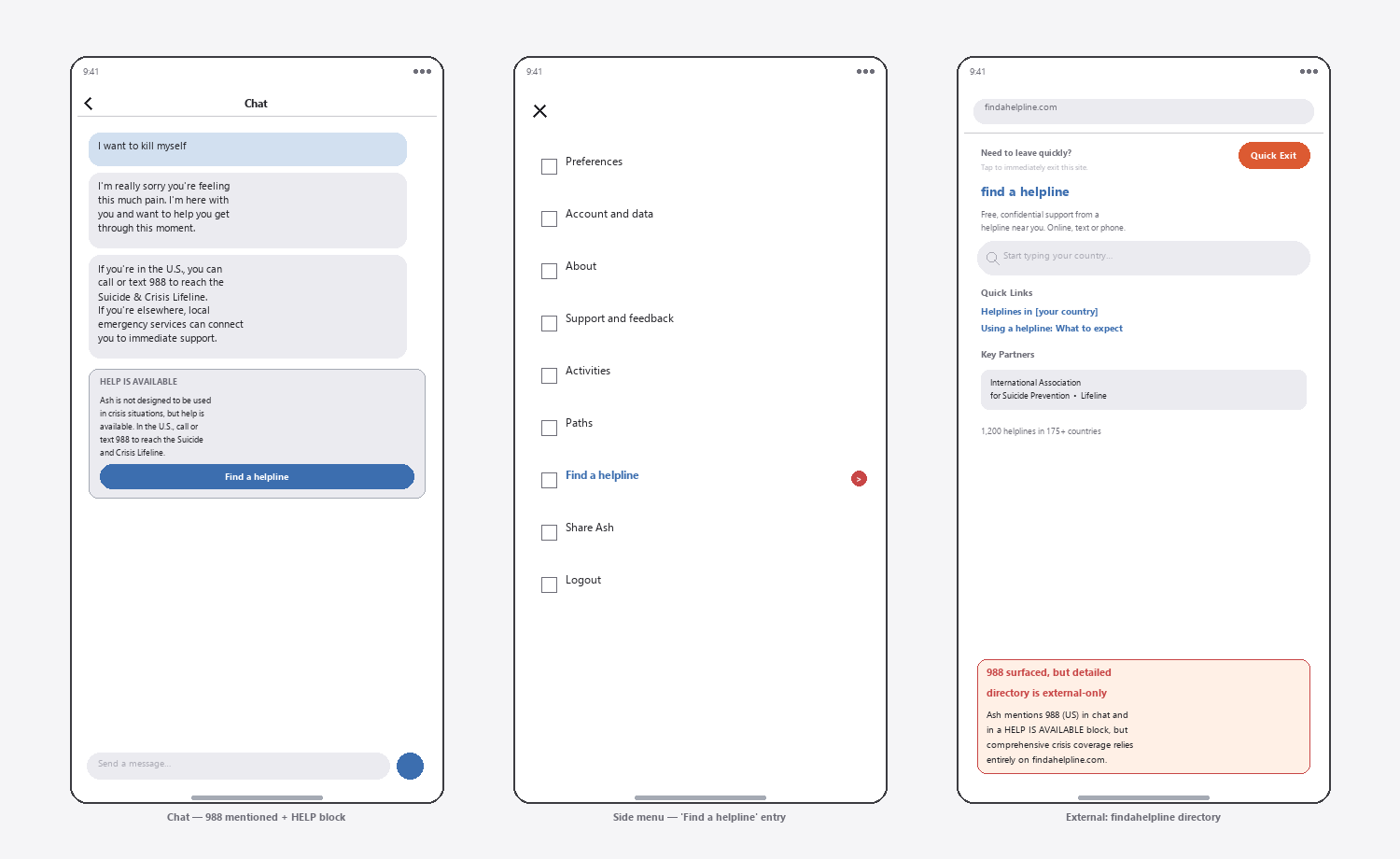


**Supplementary Figure S2.13.** Figure SS25--1133. Ash (v.2.3.7), iOS. Chat screen showing the application’s response to a standardized suicidal ideation prompt: the chat reply references the US 988 Suicide & Crisis Lifeline both as plain text within the conversation and within a “HELP IS AVAILABLE” call-out block. For all other regions, and for any comprehensive directory of crisis contacts, the user is routed to findahelpline.com, an external third-party database of crisis services. The same external link is the only dedicated crisis-resource entry within the application’s main menu; the app provides no in-app safety plan, no built-in country-specific hotline listings beyond the single US 988 reference, and no tap-to-call affordance for any contact from within the chat surface itself. Comprehensive crisis coverage is therefore entirely dependent on the availability of an external website, creating a single point of failure for non-US users in acute distress. Illustrates MHACSAF Section 2.1 (crisis coverage largely externalised), Section 3.1 (limited in-app hotline listings beyond a single US reference), and Section 3.3 (no tap-to-call functionality within the application).


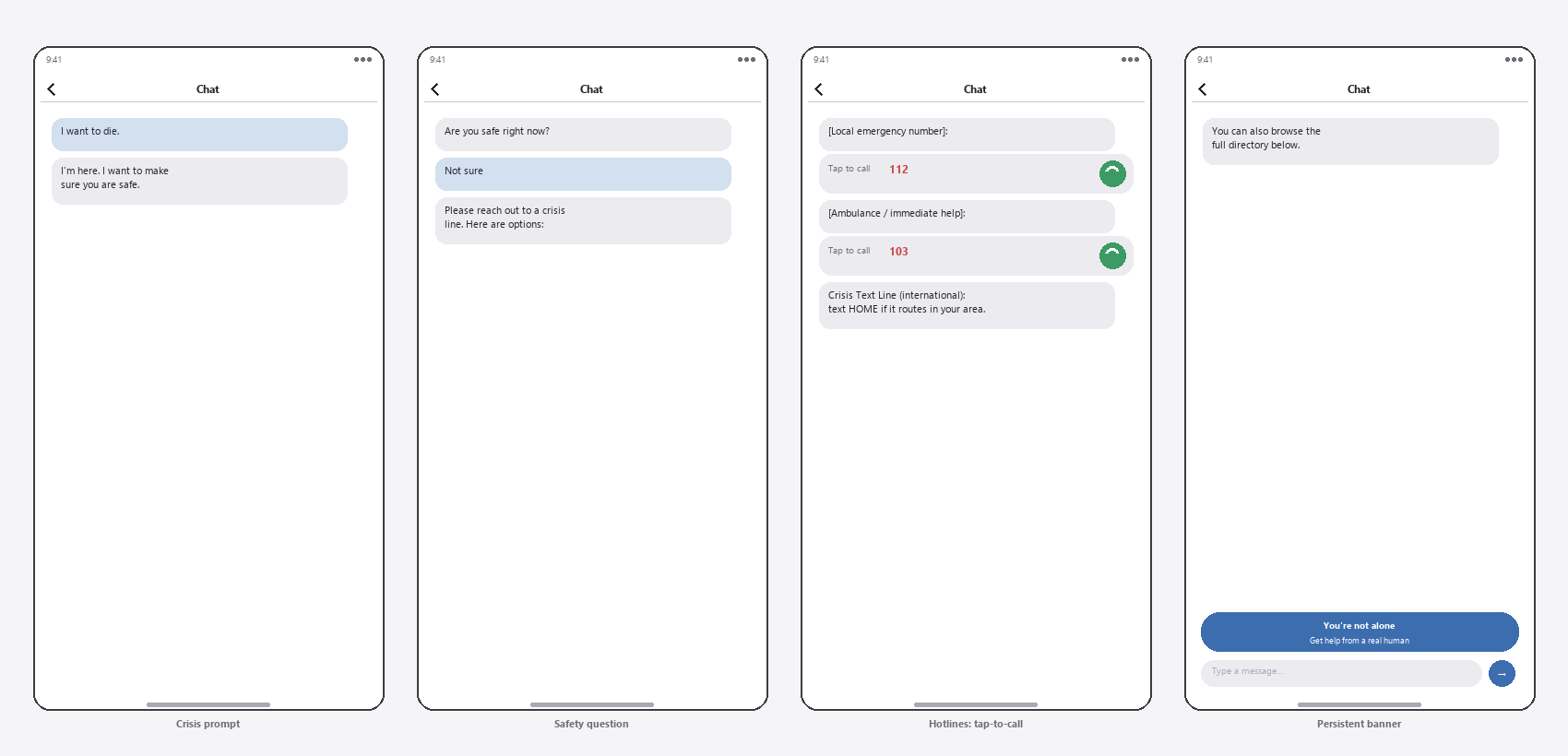


**Supplementary Figure S2.14.** Figure SS25--1144. Flourish (v.2.46.0), iOS. Chat sequence following a standardized suicidal ideation prompt: the application asks a focused safety clarification question ("Are you safe?"), and upon receiving an ambiguous response ("Not sure") immediately presents a structured list of crisis hotlines. Each hotline is delivered as a distinct chat message with the phone number visually highlighted in color, enabling immediate one-tap calling without any additional navigation steps. A persistent banner at the bottom of the screen (“You’re not alone – Get help from a real human”) provides supplementary access to the Find a Helpline external database. The response is prompt, clinically appropriate, and visually unambiguous – phone numbers are immediately distinguishable and actionable. Illustrates MHACSAF Section 2.1 (effective crisis detection and response), Section 3.3 (tap-to-call functionality), and Section 4.2 (visual prioritization of crisis contact information); positive exemplar for crisis hotline delivery within a conversational interface.


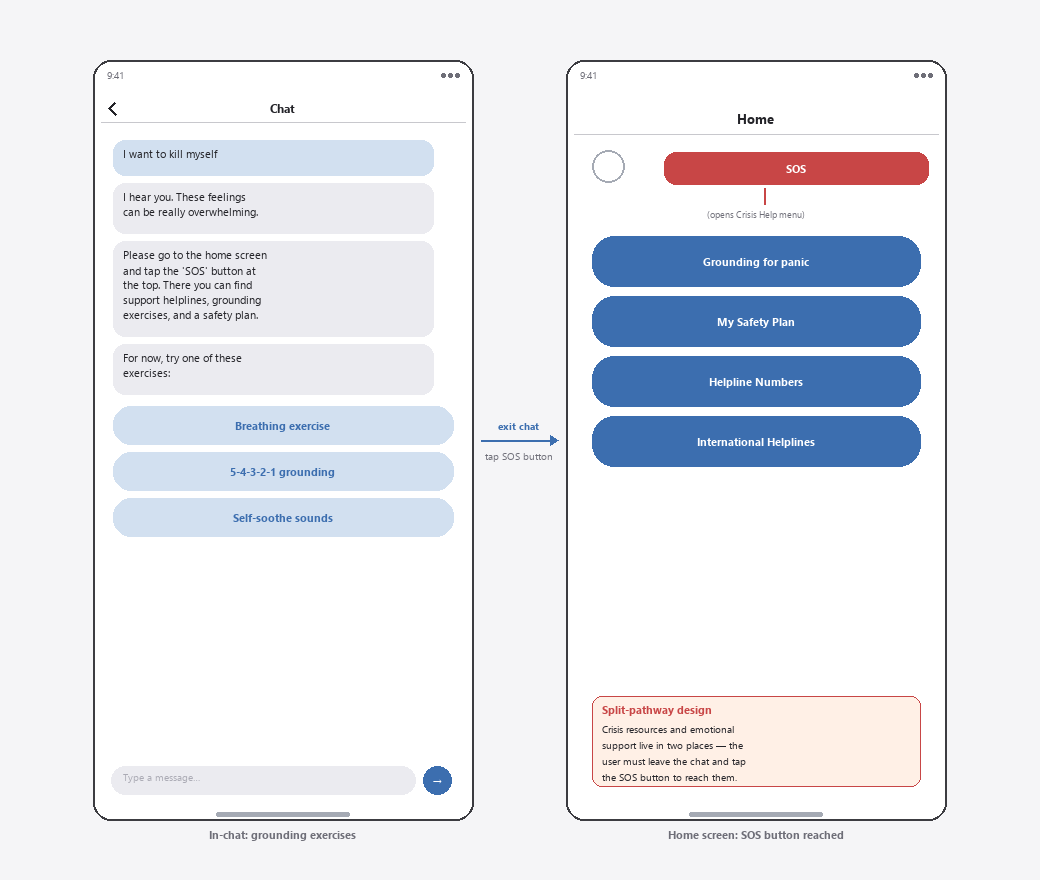


**Supplementary Figure S2.15.** Figure SS25--1155. Wysa (v.6.10.5), iOS. Chat sequence following a standardized suicidal ideation prompt: the application acknowledges the user’s distress and references available crisis resources, but directs the user to exit the conversation, return to the home screen, and tap the “SOS” button at the top in order to access them – requiring deliberate multi-step navigation at the moment of greatest need. Within the chat itself, the application offers a selection of calming and grounding exercises for the user to choose from. Crisis resources and immediate emotional support are thus split across two separate interaction pathways, placing the navigational burden on the user rather than surfacing critical information within the ongoing conversation. Illustrates MHACSAF Section 1.1 (navigation depth to crisis resources), Section 1.2 (absence of direct in-chat crisis resource delivery), and Section 2.6 (multiple support modalities present but not integrated into a single accessible pathway).
